# Intracardiac vs Transesophageal Echocardiography in Atrial Fibrillation Ablation: A Randomized Clinical Trial

**DOI:** 10.1001/jamacardio.2025.3687

**Published:** 2025-10-08

**Authors:** Xiaofeng Hu, Weifeng Jiang, Xinhua Wang, Ping Ye, Xiangting Li, Ying Wang, Qidong Zheng, Yanzhe Wang, Lihua Leng, Zengtang Zhang, Bing Han, Yu Zhang, Mu Qin, Xu Liu, Xumin Hou

**Affiliations:** 1 Department of Cardiology, Shanghai Chest Hospital, Shanghai Jiao Tong University School of Medicine, Shanghai, China; 2 Department of Cardiology, Ren Ji Hospital, Shanghai Jiao Tong University Shanghai School of Medicine, Shanghai, China; 3 Department of Cardiology, The Central Hospital of Wuhan, Tongji Medical College, Huazhong University of Science and Technology, Wuhan, China; 4 Department of Cardiology, Affiliated Hospital of Jining Medical University, Jining, China; 5 Department of Cardiology, Second Affiliated Hospital of Shandong University of Traditional Chinese Medicine, Jinan, China; 6 Department of Cardiology, Yuhuan Second People’s Hospital, Yuhuan, China; 7 Department of Cardiology, Changshu Hospital of Traditional Chinese Medicine, Changshu, China; 8 Department of Cardiology, The PLA Navy Anqing Hospital, Anqing, China; 9 Department of Cardiology, Jinan People’s Hospital, Jinan, China; 10 Department of Cardiology, Xuzhou Central Hospital, Xuzhou, China

## Abstract

**Importance:**

Transesophageal echocardiography (TEE) is the standard imaging modality for thrombus screening prior to atrial fibrillation (AF) ablation but carries procedural risks. Intracardiac echocardiography (ICE) is an alternative that may offer comparable safety with procedural advantages.

**Objective:**

To determine whether ICE is noninferior to TEE in preventing periprocedural thromboembolic events in AF ablation.

**Design, Setting, and Participants:**

This multicenter randomized clinical trial was conducted at 10 hospitals in China from August 2022 to July 2023, with a 30-day follow-up, enrolling adults with AF scheduled for catheter ablation who met predefined eligibility criteria. Data analysis was performed from August 2023 to December 2023.

**Interventions:**

Thrombus screening with ICE or TEE prior to ablation.

**Main Outcomes and Measures:**

The primary end point was the incidence of periprocedural thromboembolic events (stroke, transient ischemic attack, or systemic embolism). Secondary end points included thrombus detection, procedural safety and efficiency, and patient-reported comfort.

**Results:**

A total of 1810 patients (mean [SD] age, 64.3 [9.4] years; 868 women [48.0%]; 887 patients [49.0%] with paroxysmal AF) were randomized to ICE (n = 906) or TEE (n = 904). Thromboembolic events occurred in 4 of 906 patients undergoing ICE (0.4%) and 5 of 904 patients undergoing TEE (0.6%) (risk difference, −0.11%; Farrington-Manning 95% CI, −0.84% to 0.62%; *P* for noninferiority = .01). Thrombus was detected in 2.0% vs 1.5% (relative risk [RR], 1.29; 95% CI, 0.64-2.61; *P* = .48) with ICE vs TEE, respectively, with more non–left atrial appendage thrombi in ICE (0.6% vs 0%; *P* < .001). Major bleeding related to transseptal puncture was lower with ICE (0.2% vs 1.2%; RR, 0.18; 95% CI, 0.04-0.81; *P* = .03). ICE reduced mean (SD) fluoroscopy time (4.2 [1.5] vs 9.3 [3.0] minutes; *P* < .001), preprocedural waiting time (14.4 [8.0] vs 23.6 [10.5] hours; *P* < .001), and anxiety or depression prevalence (24.6% vs 37.5%; RR, 0.66; 95% CI, 0.56-0.76; *P* < .001).

**Conclusions and Relevance:**

In this multicenter randomized clinical trial, ICE was noninferior to TEE for preventing thromboembolic complications in AF ablation and offered additional advantages in safety, efficiency, and patient comfort, supporting its use as a viable alternative in clinical practice.

**Trial Registration:**

ClinicalTrial.gov Identifier: NCT05466266

## Key Points

**Question:**

Is intracardiac echocardiography (ICE) noninferior to transesophageal echocardiography (TEE) in preventing periprocedural thromboembolic events during atrial fibrillation (AF) ablation?

**Findings:**

In this multicenter randomized clinical trial of 1810 patients undergoing AF ablation, periprocedural thromboembolic events occurred in 4 of 906 patients in the ICE group (0.4%) and in 5 of 904 patients in the TEE group (0.6%), demonstrating noninferiority of ICE.

**Meaning:**

ICE may serve as a noninferior alternative to TEE for thromboembolic prevention during AF ablation.

## Teaser

This multicenter randomized clinical trial conducted in China determines whether intracardiac echocardiography is noninferior to transesophageal echocardiography in preventing periprocedural thromboembolic events in atrial fibrillation ablation.

## Introduction

Catheter ablation has become a first-line therapy for symptomatic atrial fibrillation (AF) in recent years. Increasing evidence also supports its role as a central component of early rhythm control strategies, which have been associated with improved cardiovascular outcomes in patients with AF.^1,2,3^Accurate detection of left atrial thrombus prior to ablation is essential for minimizing the risk of periprocedural thromboembolic events. Currently, transesophageal echocardiography (TEE) is the standard imaging modality recommended for this purpose by international guidelines.^4,5^ However, TEE is a semi-invasive procedure associated with patient discomfort and potential complications, including oropharyngeal, laryngeal, and esophageal injury.^6,7^

Intracardiac echocardiography (ICE), routinely used during AF ablation to guide catheter positioning and detect procedural complications,^8,9^ has recently gained attention for its potential in preprocedural thrombus assessment. Observational studies suggest that ICE is capable of detecting left atrial appendage (LAA) thrombus with high accuracy.^10,11,12^ Furthermore, advanced imaging techniques involving ICE catheter positioning in the pulmonary artery (PA) or right ventricular outflow tract (RVOT) may enhance visualization of the LAA and support its role as a possible alternative to TEE.^13,14^ However, ICE remains an invasive procedure that requires additional venous access and catheter manipulation and carries potential risks, including vascular injury and cardiac perforation.

Some centers perform AF ablation without preprocedural cardiac imaging, relying solely on uninterrupted anticoagulation to prevent LA thrombus; however, this strategy has limitations, as anticoagulation alone does not fully eliminate the risk of LAA thrombus, particularly in high-risk patients.^6^ Consequently, many centers continue to use cardiac imaging modalities for direct preprocedural thrombus assessment, despite ongoing anticoagulation. The prevailing consensus among guidelines is to prioritize TEE as the primary screening method, with ICE being conditionally recommended.^15,16^ Despite the latest Chinese and European Heart Rhythm Association/Heart Rhythm Society expert consensuses recommending ICE as a replacement for TEE in assessing LA thrombus, robust clinical evidence supporting this conclusion is lacking.^17,18^

To address this gap, we conducted a multicenter randomized clinical trial to evaluate the noninferiority of ICE compared to TEE not in terms of image-based thrombus detection alone, but more importantly, in terms of clinical safety as measured by the incidence of periprocedural thromboembolic events. This study aimed to provide robust evidence on the clinical applicability of ICE as a viable alternative to TEE in patients undergoing AF ablation.

## Methods

### Trial Design

This study was an investigator-initiated, multicenter, randomized, controlled, noninferiority clinical trial designed to evaluate whether ICE is noninferior to TEE in preventing periprocedural thromboembolic events among patients undergoing AF ablation across 10 tertiary hospitals in China (ClinicalTrials.gov identifier: NCT05466266). The study protocol was approved by the institutional review board of Shanghai Chest Hospital, which served as the coordinating center, and by the institutional review boards of all participating centers. The trial was conducted in accordance with the Declaration of Helsinki and relevant national regulatory requirements. The full study protocol has been published and is available in Supplement 1. Written informed consent was obtained from all patients. The study followed the Consolidated Standards of Reporting Trials (CONSORT) reporting guidelines.

### Study Participants

Participants included all adults aged 18 to 80 years undergoing AF ablation from August 2022 to July 2023 while receiving uninterrupted oral anticoagulation for at least 3 weeks, either with a direct oral anticoagulant or a vitamin K antagonist (target international normalized ratio, 2.0-3.0). Exclusion criteria were patients with contraindications to TEE, including esophageal strictures, active esophageal or gastric ulcers, significant bleeding risks, and pharyngeal malignancies or obstructive conditions. Detailed inclusion and exclusion criteria are provided in Supplement 1.

### Randomization and Allocation

Participants were randomly assigned in a 1:1 ratio to either the ICE or TEE group using a computer-generated block randomization scheme, stratified by study site to minimize center-specific variation. Randomization was coordinated through a centralized system. Due to the nature of the procedures, blinding of patients and operators was not feasible.

### Study Procedures

#### TEE Group

TEE was performed using a multiplane echocardiography system (GE Vivid E9 [GE] or iE33 [Philips]) in accordance with standard practice guidelines. Detailed data acquisition was performed at a wide range of multiplane angles (0° to 180°) and at different anatomic levels. The transducer frequency was carefully adjusted to optimize image quality. TEE was initially evaluated by an experienced echocardiographer and subsequently reviewed by at least 2 other experienced echocardiographers who were blinded to the initial TEE results. In the TEE group, transseptal puncture was performed under fluoroscopy, and all geometries were constructed with the CARTO 3 navigation system (Biosense Webster).

#### ICE Group

ICE imaging was performed using a 10F SoundStar catheter (Biosense Webster) inserted via an 11F sheath through the left femoral vein. The catheter was positioned sequentially in the right atrium, near the His bundle, in the RVOT, and the pulmonary artery to optimize LAA visualization. The ICE system was integrated with the CARTOSOUND module (Biosense Webster) for real-time 3-dimensional (3D) geometry construction, transseptal puncture guidance, and effusion monitoring. A minimal fluoroscopy strategy was applied, although zero radiation was not mandatory. Detailed procedural protocols are provided in Supplement 1.

#### Catheter Ablation

All patients received uninterrupted oral anticoagulation during the periprocedural period. Ablation procedures were performed under conscious sedation rather than general anesthesia to facilitate real-time neurological assessment and minimize the risk of cerebrovascular embolic events. Following vascular access, a bolus of heparin (100 IU/L) was administered immediately before double transseptal puncture. Anticoagulation therapy was continuously carried out throughout the procedure, targeting an activated clotting time (ACT) between 250 and 300 seconds. ACT was reassessed every 30 minutes, with additional boluses administered as needed to maintain the target ACT. Transseptal sheaths were continuously flushed with heparinized saline (2000 IU/L), 180 mL per hour. A 3D map of the LA geometry was generated using the CARTO 3 navigation system. Continuous point-by-point ablation using a 3.5-mm irrigated open-tip catheter (THERMOCOOL SMARTTOUCH Catheter D-F curve [Biosense Webster]) resulted in the formation of pulmonary vein antrum lesions in all patients. Oral anticoagulation was mandated for a minimum of 3 months following ablation. Patients in the TEE group were permitted to cross over and undergo ICE screening. All participants were followed up for a minimum of 3 months after ablation.

### Study End Points

The primary end point was the incidence of periprocedural thromboembolic events, defined as stroke, transient ischemic attack (TIA), or systemic embolism occurring within 30 days postablation. Diagnosis of stroke was confirmed by magnetic resonance imaging (MRI) or computed tomography (CT). An independent clinical events committee, blinded to group allocation, adjudicated all suspected end point events. Noninferiority was concluded if the upper limit of the 1-sided 97.5% confidence interval for the absolute difference in event rates (ICE − TEE) did not exceed the prespecified noninferiority margin of 0.8%.

Secondary end points, evaluated within 30 days of ablation, included thrombus detection rate, all-cause mortality, major bleeding complications (eg, cardiac tamponade, pericardial effusion), minor bleeding events (eg, vascular complications, such as pseudoaneurysms or hematomas), procedural characteristics (eg, fluoroscopy time, ablation time), and preprocedural waiting time. A full list of secondary end points is provided in Supplement 1. All safety-related clinical end points were adjudicated by an independent clinical event committee, while overall study oversight and interim safety monitoring were conducted by a separate data and safety monitoring board.

### Statistical Analysis

Continuous variables were summarized as mean with standard deviation or median with interquartile range, depending on distribution, and compared using the *t* test or Mann-Whitney *U* test. Categorical variables were presented as numbers and percentages and compared with χ^2^ or Fisher exact tests. All analyses followed the intention-to-treat (ITT) principle. For the noninferiority design, parameters were 1-sided α = .025, β = 0.20, expected event rates of 0.6% (ICE) and 0.5% (TEE), and a noninferiority margin of 0.8%, yielding a required sample size of 1750. The final enrollment of 1810 patients provided slightly greater power than the target 80%. The primary end point was evaluated using risk difference (RD) with Farrington-Manning 95% confidence intervals, and noninferiority was concluded if the upper confidence interval limit was below the prespecified margin. Binary secondary end points were reported as both RD and relative risk (RR) with 95% confidence intervals; continuous end points were expressed as mean difference (MD) with 95% confidence intervals. All statistical tests were 2-sided, with significance set at *P* < .05, except for the 1-sided noninferiority test. Analyses were performed with SAS version 9.4 (SAS Institute).

## Results

### Patients

From August 2022 to July 2023, 1832 patients were screened and 1810 were randomized to TEE (n = 904) or ICE (n = 906) (Figure). The mean (SD) patient age was 64.3 (9.4) years; 923 patients (51.0%) had persistent or long-standing persistent AF, 868 patients (48.0%) were female, and the mean (SD) CHA_2_DS_2_-VASc score was 2.1 (1.4). Baseline characteristics were well balanced between groups (Table 1). In the TEE group, 887 patients completed the examination, and 17 patients could not tolerate esophageal intubation and crossed over to ICE. ICE imaging was successful in all assigned patients. Because thrombus detection precluded ablation in 18 ICE and 14 TEE patients, the modified ITT (mITT) population included 888 and 890 patients undergoing ICE and TEE, respectively. The per-protocol population comprised 905 and 873 patients undergoing ICE and TEE, respectively. Baseline characteristics for the mITT and per-protocol analyses are provided in eTables 1 and 2 in Supplement 2.

**Figure.**
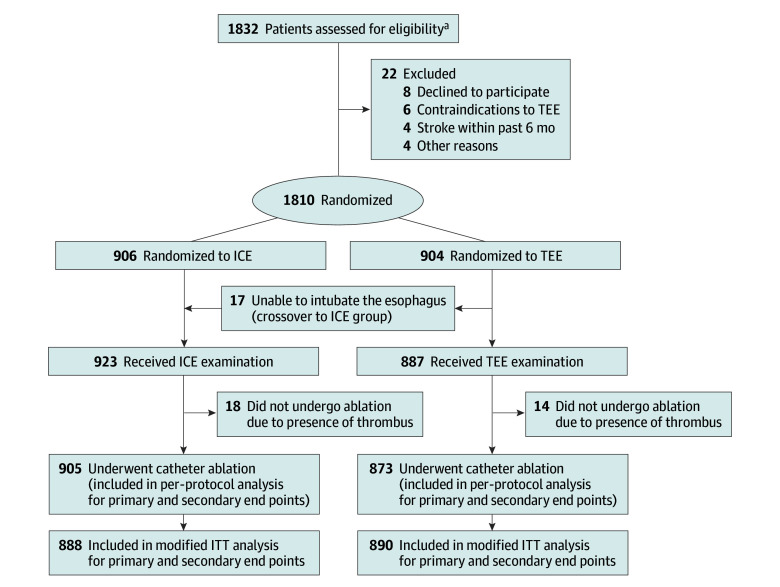
CONSORT Flow Diagram ICE indicates intracardiac echocardiography; ITT, intention-to-treat; TEE, transesophageal echocardiography. ^a^Ten centers were not required to provide screening logs during the recruitment phase; thus, the number of patients for eligibility assessment is not available.

**Table 1.** Baseline Characteristics of Study Patients

Characteristic	No. (%)	
	ICE group (n = 906)	TEE group (n = 904)
Age, mean (SD), y	64.4 (9.4)	64.2 (9.5)
Sex		
Female	428 (47.2)	440 (48.7)
Male	478 (52.8)	464 (51.3)
BMI, mean (SD)^a^	23.1 (2.1)	23.0 (2.1)
Smoking status	247 (27.3)	262 (29.0)
Drinking	265 (29.3)	279 (30.9)
AF type		
Paroxysmal AF	459 (50.7)	428 (47.3)
Persistent AF	312 (34.4)	321 (35.5)
Long-standing AF	135 (14.9)	155 (17.2)
CHA_2_DS_2_-VASc score, mean (SD)	2.2 (1.4)	2.1 (1.3)
Concomitant disease		
Hypertension	546 (60.3)	538 (59.5)
Prior stroke or TIA	92 (10.2)	89 (9.8)
Coronary artery disease	204 (22.5)	181 (20.0)
Diabetes	145 (16.0)	155 (17.1)
Heart failure	31 (3.4)	24 (2.7)
Echocardiographic findings		
LAD, mean (SD), mm	43.2 (5.0)	43.4 (4.9)
LVEF, mean (SD), %	62.6 (4.3)	62.7 (3.7)
Anticoagulant regimen		
Rivaroxaban	863 (95.3)	850 (94.0)
Dabigatran	35 (3.9)	47 (5.2)
Vitamin K antagonist	8 (0.9)	7 (0.8)

Abbreviations: AF, atrial fibrillation; BMI, body mass index; ICE, intracardiac echocardiography; LAD, left atrial diameter; LVEF, left ventricle ejection fraction; TEE, transesophageal echocardiography; TIA, transient ischemic attack.

^a^ Calculated as weight in kilograms divided by height in meters squared.

### End Points

#### Primary End Points

All patients underwent pulmonary vein isolation as the foundational ablation strategy, with additional lesion sets (posterior wall, mitral isthmus, cavotricuspid isthmus, or superior vena cava isolation) performed in similar proportions between the ICE and TEE groups (eTable 3 in Supplement 2). Periprocedural thromboembolic events (from vascular puncture to 30 days) occurred in 4 patients (0.4%) in the ICE group (1 stroke, 3 TIAs) and 5 patients (0.6%) in the TEE group (all TIAs), with no statistically significant difference between groups (Table 2). The absolute risk difference (ICE − TEE) was −0.11% (95% CI, −0.65% to 0.43%), and the upper bound of the 1-sided 97.5% confidence interval was below the prespecified noninferiority margin of 0.8%, meeting the criterion for noninferiority. Primary end point results for the mITT and per-protocol populations are shown in eTables 4 and 5 in Supplement 2. Detailed clinical information for all 9 patients with thromboembolic events is provided in eTable 6 in Supplement 2.

**Table 2.** Procedural and Clinical Outcomes

End point	No. (%)		Treatment effect (95% CI)^a^	*P* value
	ICE group (n = 906)	TEE group (n = 904)		
Thromboembolic events	4 (0.4)	5 (0.6)		
Stroke	1 (0.1)	0	−0.11% (−0.84% to 0.62%)	.01^b^
TIA	3 (0.3)	5 (0.6)		
Systemic embolism	0	0		
Presence of thrombus	18 (2.0)	14 (1.5)		
LAA	13 (1.4)	14 (1.5)	1.29 (0.64-2.61)	.48
Non-LAA	5 (0.6)	0		
Death	0	0	NA	NA
Major bleeding events	7 (0.8)	16 (1.8)		
Pericardial effusions	5 (0.6)	7 (0.8)	0.43 (0.18-1.06)	.07
Cardiac tamponade requiring pericardiocentesis or surgery	2 (0.2)	9 (1.0)		
Major bleeding related with transseptal puncture	2 (0.2)	11 (1.2)	0.18 (0.04-0.81)	.03
Minor bleeding events	12 (1.3)	10 (1.1)		
Groin hematoma	8 (0.9)	7 (0.8)	1.00 (0.42-2.42)	.99
Pseudoaneurysm	2 (0.2)	3 (0.3)		
Arteriovenous fistulas	0	0		
Total procedure time, mean (SD), min	159.6 (25.4)	144.9 (24.8)	14.7 (12.6 to 16.8)	<.001
Radiofrequency delivery time, mean (SD), min	56.4 (10.7)	56.0 (11.7)	0.4 (−0.5 to 1.3)	.38
Fluoroscopy time, mean (SD), min	4.2 (1.5)	9.3 (3.0)	−5.1 (−5.4 to −4.8)	<.001
Preprocedural waiting time, mean (SD), h	14.4 (8.0)	23.6 (10.5)	−9.2 (−10.1 to −8.3)	<.001

Abbreviations: ICE, intracardiac echocardiography; LAA, left atrial appendage; NA, not applicable; TEE, transesophageal echocardiography; TIA, transient ischemic attack.

^a^ Treatment effect is presented as risk difference or relative risk with 95% confidence intervals for binary outcomes and as mean difference with 95% confidence intervals for continuous outcomes.

^b^ *P* for noninferiority.

The stroke case in the ICE group involved a patient with persistent AF (CHA_2_DS_2_-VASc score: 3) who underwent pulmonary vein isolation and driver ablation, achieving sinus rhythm after direct-current cardioversion. The next day, the patient developed right hand weakness. Head CT was negative, but brain MRI revealed acute infarcts in the left frontal and right occipital lobes (eFigure 1 in Supplement 2).

#### Secondary End Points

##### Thrombus Detection Rate

Clear definition and complete visualization of the LA and LAA were achieved in all patients in both groups (different imaging modalities for LAA observation are shown in eFigure 2 in Supplement 2). Thrombus was detected in 18 patients (2.0%) in the ICE group and 14 patients (1.5%) in the TEE group (RR, 1.29; 95% CI, 0.64-2.61; *P* = .48) (Table 2). In the ICE group, 5 patients had thrombi located outside the LAA—1 in the LA roof, 2 in the ridge of the left pulmonary vein, 1 in the interatrial septum, and 1 on the pulmonary valve (representative case shown in eFigure 3 in Supplement 2). Among patients with thrombus, the proportion of non-LAA thrombi was significantly higher in the ICE group compared with the TEE group (27.8% vs 0%; *P* = .03).

##### Procedure-Related Complications

Procedure-related complications are summarized in Table 2. Major bleeding occurred in 7 patients (0.8%) in the ICE group and 16 patients (1.8%) in the TEE group (RR, 0.43; 95% CI, 0.18-1.06; *P* = .07). Major bleeding related to transseptal puncture was significantly less frequent with ICE than with TEE (0.2% vs 1.2%; RR, 0.18; 95% CI, 0.04-0.81; *P* = .03). Minor bleeding occurred in 12 patients (1.3%) in the ICE group and 10 patients (1.1%) in the TEE group (RR, 1.00; 95% CI, 0.42-2.42; *P* = .99). No deaths were reported in either group. Complication data for the mITT and per-protocol populations are provided in eTables 7 and 8 in Supplement 2.

##### Procedural Characteristics

As shown in Table 2, the radiofrequency delivery time was comparable between the TEE and ICE groups (MD, 0.4 minutes; 95% CI, −0.5 to 1.3; *P* = .38). The total procedure time was significantly longer in the ICE group (MD, 14.7 minutes; 95% CI, 12.6-16.8; *P* < .001), whereas fluoroscopy time was significantly shorter (MD, −5.1 minutes; 95% CI, −5.4 to −4.8; *P* < .001).

##### Preprocedural Psychological State and Procedural Pain

As shown in Table 3, Hospital Anxiety and Depression Scale (HADS) anxiety and depression scores were significantly lower in the ICE group compared with the TEE group (anxiety: MD, −0.6; 95% CI, −1.0 to −0.2; *P* < .001; depression: MD, −0.6; 95% CI, −0.9 to −0.3; *P* < .001). Clinically relevant anxiety and/or depression occurred in 24.6% vs 37.5% of patients (RR, 0.66; 95% CI, 0.56-0.76; *P* < .001) in the ICE and TEE groups, respectively. Procedural pain incidence was markedly lower with ICE (1.0% vs 89.6%; *P* < .001), with a median (IQR) Numeric Rating Scale 11 score of 0 (0-0) vs 2 (1-3). Postprocedural discomfort was reported in 225 patients undergoing TEE (24.9%) but in none of the patients undergoing ICE.

**Table 3.** Patient-Reported Outcomes

End point	No. (%)		Treatment effect (95% CI)^a^	*P* value
	ICE group (n = 906)	TEE group (n = 904)		
HADS anxiety score, mean (SD)	6.3 (4.5)	6.9 (4.5)	−0.6 (−1.0 to −0.2)	<.001
HADS depression score, mean (SD)	5.7 (3.8)	6.3 (3.8)	−0.6 (−0.9 to −0.3)	<.001
Anxiety and depression state	223 (24.6)	339 (37.5)	0.66 (0.56 to 0.76)	<.001
Anxiety state	218 (24.1)	336 (37.2)	0.65 (0.56 to 0.76)	<.001
Depression state	203 (22.4)	300 (33.2)	0.68 (0.57 to 0.80)	<.001
Procedural pain (NRS11 score), median (IQR)	0 (0-0)	2 (1-3)	NA	<.001
NRS11 score category				
No pain	897 (99.0)	94 (10.3)	9.62 (8.04 to 11.51)	<.001
Mild pain	9 (1.0)	729 (80.6)	0.01 (0.01 to 0.02)	<.001
Moderate pain	0	81 (9.0)	NA	<.001
Severe pain	0	0	NA	NA
Postprocedural pain or discomfort	NA	225 (24.9)	NA	NA

Abbreviations: HADS, Hospital Anxiety and Depression Scale; ICE, intracardiac echocardiography; NA, not applicable; NRS11, Numeric Rating Scale 11; RR, relative risk; TEE, transesophageal echocardiography.

^a^ Treatment effect is presented as mean difference for continuous outcomes and as relative risk for binary outcomes.

##### Procedural Efficiency and Hospitalization Cost

The mean (SD) preprocedural waiting time was significantly shorter in the ICE group compared with the TEE group (14.4 [8.0] hours vs 23.6 [10.5] hours; MD, −9.2 hours; 95% CI, −10.1 to −8.3; *P* < .001) (Table 2). After adjusting for inflation, the total mean (SD) hospitalization cost for patients undergoing AF ablation was significantly higher in the ICE group compared with the TEE group (¥101 606 [¥15 170] [US $14 199.20 ($2119.98)] vs ¥84 575 [¥11 920] [US $11 819.20 ($1665.80)]; MD, ¥17 031; 95% CI, ¥15 093-¥18 969 [MD, US $2380.05; 95% CI, $2109.22-$2650.88]; *P* < .001). However, because all procedures were covered by the national medical insurance system, the actual out-of-pocket expenses for patients increased only slightly.

##### Subgroup Analysis

Prespecified subgroup analyses of the primary end point were conducted across 6 predefined categories (eTable 9 in Supplement 2). No statistically significant interaction between subgroup factors and treatment assignment (ICE vs TEE) was observed.

## Discussion

This multicenter randomized clinical trial is the first to prospectively evaluate whether ICE can safely and effectively replace TEE for thrombus screening prior to AF ablation. Notably, the incidence of stroke was low and comparable between the 2 groups. Our findings demonstrate that ICE achieves noninferiority to TEE in preventing periprocedural thromboembolic events through equivalent efficacy in thrombus screening. In addition, ICE offers multiple procedural and safety advantages, including the detection of non-LAA thrombi, a lower incidence of major bleeding complications, and improved patient comfort and workflow efficiency. These results provide robust clinical evidence to support the broader adoption of ICE as a first-line imaging modality in selected patients undergoing AF ablation.

Although periprocedural thromboembolic events are relatively infrequent, they remain among the most serious complications of AF catheter ablation, primarily resulting from undetected preexisting LA thrombi or suboptimal anticoagulation management.^19^ While uninterrupted anticoagulation can reduce the incidence of periprocedural thromboembolic events to approximately 0.4% to 0.7%,^20^ some centers have adopted a strategy of omitting routine preprocedural imaging in adequately anticoagulated low-risk patients. However, this practice is primarily based on observational data and lacks support from randomized clinical trials. Moreover, recent meta-analyses have demonstrated that the risk of LA or LAA thrombus remains non-negligible even after 3 or more weeks of therapeutic anticoagulation, with a weighted mean prevalence of 2.73%, increasing to 4.81% in patients with nonparoxysmal AF and 6.31% in those with CHA_2_DS_2_-VASc scores of 3 or higher.^6^ These findings underscore that anticoagulation alone does not reliably eliminate the risk of thrombus, particularly in high-risk populations. Supporting this, several pivotal randomized clinical trials—including RE-CIRCUIT, ELIMINATE-AF, ASCERTAIN, and AXAFA-AFNET 5—reported extremely low thromboembolic event rates (0%-0.3%), yet nearly all participants underwent systematic preprocedural imaging.^21,22,23,24^ Specifically, in RE-CIRCUIT, ELIMINATE-AF, and ASCERTAIN, 100% of patients received TEE or ICE, while 86.7% of patients in AXAFA-AFNET 5 underwent TEE despite uninterrupted apixaban therapy. These data collectively suggest that favorable outcomes in these trials reflect the combined effect of adequate anticoagulation and systematic thrombus exclusion rather than anticoagulation alone. In our study, a similarly low rate of periprocedural thromboembolic events was observed, likely due to the dual strategy of consistent anticoagulation and structured thrombus screening via TEE or ICE. Although LA thrombus detection and actual embolic events are not equivalent, they are closely related in terms of patient safety; thus, preprocedural imaging remains essential—even in adequately anticoagulated patients—and should be maintained as a key component of AF ablation.

Several imaging modalities, including TEE, ICE, and delayed-phase cardiac CT (CCT), are available for the exclusion of atrial thrombus prior to AF ablation. Although TEE is widely regarded as the criterion standard for preprocedural thrombus screening, it has several limitations in routine clinical practice. TEE requires esophageal intubation and often requires sedation, which may reduce patient tolerance and limit its applicability in certain populations. Moreover, due to anatomical constraints of the esophagus, imaging angles may be restricted, resulting in suboptimal or inconclusive visualization of the LAA; up to 17.8% of TEE examinations have been reported to yield inadequate image quality, potentially delaying clinical decision-making or necessitating repeat imaging.^12,25^ In contrast, ICE offers several technical and operational advantages that may overcome these shortcomings. When positioned in the PA, ICE enables near-field, high-resolution imaging of the LAA from multiple angles, allowing comprehensive assessment of fine anatomical details, including pectinate muscles, thrombus borders, and their spatial relationships with the LAA wall.^10,26^ In addition, ICE allows operator-controlled, real-time probe manipulation, offering greater flexibility and adaptability—capabilities not achievable with TEE.^10,11,12^ In our study, all patients in the ICE group successfully underwent LAA imaging without the need for crossover or additional imaging, and ICE detected thrombi in atypical locations not identified by TEE. Compared with TEE and ICE, delayed-phase CCT offers a noninvasive alternative for imaging-based exclusion of atrial thrombus. A recent meta-analysis of 27 studies^27^ demonstrated that delayed-phase CCT provides high diagnostic accuracy and may serve as a reliable substitute for TEE. However, its potential for false-positive findings should be acknowledged. In cases with equivocal or positive CCT results, confirmatory TEE may still be warranted to ensure diagnostic accuracy and avoid unnecessary delays in ablation procedures.^28^ Overall, ICE represents a flexible and efficient imaging modality that may serve as a viable alternative to TEE in a variety of real-world clinical scenarios, particularly with respect to image clarity, maneuverability, and patient suitability.

Beyond safety and diagnostic accuracy, ICE offers significant advantages in procedural safety and patient experience. Notably, ICE was associated with a significantly lower incidence of major bleeding complications related to transseptal puncture compared to TEE, suggesting a potential safety benefit when ICE is used to guide transseptal access. This may reflect improved spatial orientation and real-time visualization of interatrial septal anatomy afforded by ICE imaging. Furthermore, ICE obviates the need for esophageal intubation, sedation, and additional anesthesia preparation, thereby shortening preprocedural wait times and simplifying workflow. In our study, the average wait time before ablation was significantly shorter in the ICE group, with more than one-third of patients in the ICE group undergoing ablation on the day of admission. Psychological assessments also favored ICE, with lower preprocedural anxiety and depression scores, less intraprocedural pain, and fewer postprocedural complaints, highlighting the importance of patient-centered considerations in elective AF ablation. However, ICE is an invasive technique requiring additional venous access and specialized operator expertise. While vascular complication rates were slightly higher than TEE in our study, the difference was not statistically significant. Its broader adoption may be limited by training demands and higher procedural costs, especially in centers without electrophysiology specialization.

Although ICE is currently only conditionally recommended in clinical guidelines for pre-ablation thrombus screening in AF, such recommendations are largely based on observational studies and expert consensus, with a lack of high-quality randomized clinical trial data.^13,17,18,29^ In a recent survey of AF guideline writing committee members, most (59%) reported routinely using TEE for thrombus exclusion prior to ablation, followed by CCT (23%) and ICE (18%).^18^ While ICE is increasingly used for procedural guidance during AF ablation, its role in thrombus screening remains underrecognized in clinical practice. Addressing this evidence gap, our study provides the first outcome-driven, randomized clinical trial evidence demonstrating that ICE is a clinically feasible and safe alternative to TEE for preprocedural thrombus exclusion in patients undergoing AF ablation.

### Limitations

First, the open-label design may have introduced bias in the assessment of subjective secondary outcomes, including patient-reported experience and procedural workflow, which were not evaluated in a blinded manner. Second, silent cerebral embolic events were not systematically assessed using neuroimaging, potentially underestimating the true incidence of periprocedural thromboembolism. Third, all ablation procedures were performed with radiofrequency energy, which may limit generalizability to other ablation modalities, such as cryoballoon or pulsed field ablation.

## Conclusions

In this multicenter randomized clinical trial, ICE was noninferior to TEE for preprocedural thrombus screening in patients undergoing AF ablation, providing comparable protection against thromboembolic events while demonstrating advantages in procedural safety, patient comfort, and workflow efficiency.

## Notes

Supplement 1. Trial Protocol

Supplement 2. eTable 1. Baseline Characteristics of Study Patients (Modified Intention-to-Treat) eTable 2. Baseline Characteristics of Study Patients (Per-Protocol Set) eTable 3. Comparison of Ablation Strategies Between the ICE and TEE Groups eTable 4. Primary End Points (Modified Intention-to-Treat) eTable 5. Primary End Points (Per-Protocol Set) eTable 6. Baseline Characteristics of Patients With Stroke or Transient Ischemic Attack eTable 7. Secondary End Points (Modified Intention-to-Treat) eTable 8. Secondary End Points (Per-Protocol Set) eTable 9. Primary End Point Subgroup by ITT Analysis eFigure 1. Diffusion-Weighted MRI Images Showing Acute Ischemic Stroke Following AF Ablation in an ICE Group Patient eFigure 2. Representative ICE and TEE Images of the Left Atrial Appendage From Different Acoustic Windows eFigure 3. Representative Images of Non–Left Atrial Appendage Thrombi Identified by ICE

Supplement 3. ICE vs TEE Study Investigators

Supplement 4. Data Sharing Statement
