# Supplementary material for: Intracardiac vs Transesophageal Echocardiography in Atrial Fibrillation Ablation: A Randomized Clinical Trial
Source: JAMA Cardiol. 2025 Oct 8;10(12):1249–56. doi: 10.1001/jamacardio.2025.3687 (PMC12509080; doi:10.1001/jamacardio.2025.3687)
Supplement: Supplement 1. — Trial Protocol [file jamacardiol-e253687-s001.pdf]

Comparison of Peri-procedural Complications of Intracardiac Echocardiography and Transesophageal Echocardiography in Patients With Atrial Fibrillation (ICE VS TEE Study)

Study Protocol

Version 3.1, July 10, 2022

NCT05466266

Principal investigator

Name: Xu Liu
Hospital: Shanghai Chest Hospital
Address：No. 241 West Huaihai Road, Xuhui District
City：Shanghai
Country：China
Telephone：+86 18101817225
E-mail：drliuxu@126.com

Table of Contents

1.Trial information ... 3

1.1 Title ... 3

1.2 Trial Registration ... 3

1.3 Protocol Version and Date ... 4

1.4 Funding and Support ... 5

1.5 Roles and Responsibilities ... 5

2. Introduction ... 6

2.1 Background and Rationale ... 6

2.2 Objectives ... 7

2.3 Trial Design ... 7

3. Methods: Participants, Sample size justification , Interventions, and Endpoints ... 8

3.1 Study Setting ... 8

3.2 Sample size justification ... 8

3.3 Eligibility Criteria ... 9

3.4 Interventions ... 10

3.5 Endpoints ... 12

4. Methods: Assignment of Interventions ... 14

4.1 Allocation ... 14

4.2 Blinding (Masking) ... 14

4.3 Implementation ... 14

5. Methods: Data Collection, Management, Quality Control and Analysis ... 15

5.1 Data Collection Methods 15
5.2 Data Management 16
5.4 Quality Control 16
5.4 Statistical Methods 17
6. Methods: Monitoring 18
6.1 Data Monitoring 18
6.2 Auditing 19
7. Ethics and Dissemination 19
7.1 Research Ethics Approval 19
7.2 Protocol Amendments 20
7.3 Informed Consent 20
7.4 Confidentiality 21
7.5 Declaration of Interests 21
7.6 Access to Data 21
7.7 Ancillary and Post-Trial Care 21
8. Protocol modifications 21
8.1 Eligibility Criteria Modification 21
8.2 Primary Study Endpoints Modification 22
8.3 Planned Sample Size Adjustment 22
8.4 Additional Secondary Endpoints 22
8.5 Study Centers Change 22
9. Appendices 23

9.1 Appendix A: List of Abbreviations **23**

9.2 Appendix B: References **23**

1. Trial Information

1.1 Title

Comparison of Peri-procedural Complications of Intracardiac Echocardiography and Transesophageal Echocardiography in Patients With Atrial Fibrillation (ICE vs TEE Study): A Multicenter Randomized Controlled Trial

1.2 Trial Registration

- Primary Registry: ClinicalTrials.gov
- Trial Identifying Number: NCT05466266
- Date of Registration: 2021-12-15
- Scientific Title: Comparison of Peri-procedural Complications of Intracardiac Echocardiography and Transesophageal Echocardiography in Patients With Atrial Fibrillation (ICE vs TEE Study)
- Public Title: ICE vs TEE Study
- Countries of Recruitment: China
- Health Conditions Studied: Atrial Fibrillation
- Intervention(s):

    1. ICE group: Intracardiac echocardiography for LAA thrombus screening
    2. TEE group: Transesophageal echocardiography for LAA thrombus screening

- Study Type: Multicenter, randomized, open-label, parallel-group, non-inferiority trial
- Target Sample Size: 1810 participants
- Start Date of Enrollment: August 2022

1.3 Protocol Version and Date

- Protocol Version: Version 3.1
- Date: July 10, 2022

1.4 Funding and Support

- This study is funded by the Biosense Webster Inc. USA.
- The sponsor had no role in the study design, data collection, analysis, interpretation, manuscript writing, or publication decisions.
- This is an investigator-initiated and investigator-conducted study.

1.5 Roles and Responsibilities

Principal Investigator

- Name: Dr. Xu Liu
- Institution: Department of Cardiology, Shanghai Chest Hospital, School of Medicine, Shanghai Jiao Tong University
- Address: No. 241 West Huaihai Road, Xuhui District, Shanghai, China
- Email: drliuxu@126.com
- Phone: +86 18101817225

Trial Governance Structure

- Steering Committee: Oversees scientific conduct and governance
- Data Safety Monitoring Board (DSMB): An independent committee responsible for ongoing safety surveillance, periodic review of accumulated safety and efficacy data, and recommendations regarding study continuation, modification, or termination. The DSMB operates under a predefined charter and is independent from the sponsor and investigators.
- Clinical Endpoint Committee (CEC): An independent, blinded panel responsible for reviewing and adjudicating all relevant safety and efficacy endpoints to ensure objective outcome assessment.
- Data Management Team: Responsible for centralized data processing using an electronic data capture system; each site retains medical records and procedural data
- Contract Research Organization (CRO): Provides routine site monitoring and ensures regulatory compliance
- Ethics Review: Conducted per Declaration of Helsinki, ISO 14155, and local ethical requirements. All protocol amendments and SAE reports are submitted to relevant IRBs/Ecs

2. Introduction

2.1 Background and Rationale

Atrial fibrillation (AF) is the most common sustained cardiac arrhythmia, characterized by rapid and irregular atrial electrical activity. This disrupts effective atrial contraction and hemodynamics, leading to blood stasis in the left atrial appendage (LAA) and significantly increasing the risk of thrombus formation. Studies have shown that the incidence of LAA thrombi in anticoagulated patients with AF ranges from 0.4% to 15.2%, depending on individual characteristics and comorbidities.[1] The presence of LAA thrombus is considered an absolute contraindication to catheter ablation.[2] Once detected, the procedure must be postponed until adequate anticoagulation therapy has resolved the thrombus.

Transesophageal echocardiography (TEE) has long been regarded as the gold standard for preprocedural LAA thrombus screening, with a reported sensitivity and specificity exceeding 90%. [3] [4] TEE enables high-resolution imaging by positioning an ultrasound probe in the esophagus, close to the heart. In a global survey of AF ablation centers, 73.2% reported routine use of TEE before ablation, regardless of patients' CHA_2DS_2-VASc scores. [5] However, despite its diagnostic accuracy, TEE is semi-invasive and associated with discomfort and procedural risks such as gag reflex, sedation-related complications, and even esophageal injury.[6] Additionally, when image quality is suboptimal, the sensitivity of TEE to exclude LAA thrombi is reduced. The proportion of equivocal TEE findings has been reported to be as high as 17.8%, leading to potential misdiagnosis and procedural delays. [7]

Intracardiac echocardiography (ICE) is routinely used during AF ablation procedures for transseptal puncture guidance and complication monitoring. Emerging evidence suggests that ICE, when performed from appropriate positions such as the pulmonary artery or right ventricular outflow tract, may allow for reliable detection of LAA thrombi. Studies by Di Biase et al., involving over 7,000 patients undergoing uninterrupted anticoagulation and AF ablation, demonstrated the safety and feasibility of using ICE as an alternative to TEE for preprocedural thrombus screening. [8] [9] A recent retrospective study also showed that ICE provides comparable thrombus detection and complication rates, with added advantages such as lower post-procedural fever and improved hospital efficiency. [10] However, these findings are derived from retrospective or non-randomized studies, limiting the strength of the evidence.

In the ICE-CHIP trial, ICE showed lower sensitivity for LAA thrombus detection compared to TEE, likely due to the limited imaging range as the catheter remained in the right atrium.[11] With advancements in imaging technology and catheter positioning, ICE can now offer high-resolution visualization of the entire LAA, enabling its use as a potential replacement for TEE. While recent consensus documents from EHRA/HRS and China acknowledge the potential of ICE to replace TEE in LAA thrombus assessment, the recommendation remains weak due to the lack of randomized controlled evidence. [12] [13]

Therefore, we designed this prospective, multicenter, randomized controlled trial to systematically evaluate the diagnostic performance, safety, and clinical utility of ICE versus TEE in LAA thrombus screening before AF ablation. This study aims to fill the current evidence gap and provide high-level clinical data to support guideline recommendations and optimize procedural workflows.

In this study, **periprocedural embolic events**—including stroke, transient ischemic attack (TIA), and systemic embolism—were selected as the **primary endpoint**, rather than the thrombus detection rate. This decision was based primarily on **patient-centered clinical decision-making**. In real-world practice, the purpose of preprocedural screening is not merely to identify thrombi but to prevent actual thromboembolic complications during or after ablation. Hard endpoints such as stroke more directly reflect the clinical effectiveness of different strategies, whereas thrombus presence is only a surrogate marker. Thus, using embolic events as the primary endpoint enhances both the interpretability and real-world applicability of the findings, ultimately improving patient care.

2.2 Objectives

The primary objective of this study is to evaluate whether ICE is non-inferior to TEE in excluding LA or LAA thrombus prior to AF ablation. By accurately identifying patients without thrombus, the study aims to determine whether ICE can serve as a safe and effective alternative imaging modality, potentially reducing thrombus-related procedural complications and improving procedural workflow.

2.3 Trial Design

This study is a multicenter, prospective, randomized, open-label, parallel-group, non-inferiority trial. A total of 1810 patients undergoing AF ablation were randomized 1:1 to receive either ICE-guided or TEE-guided thrombus screening. Outcome assessors, including the Clinical Endpoint Committee (CEC), are blinded to group allocation.

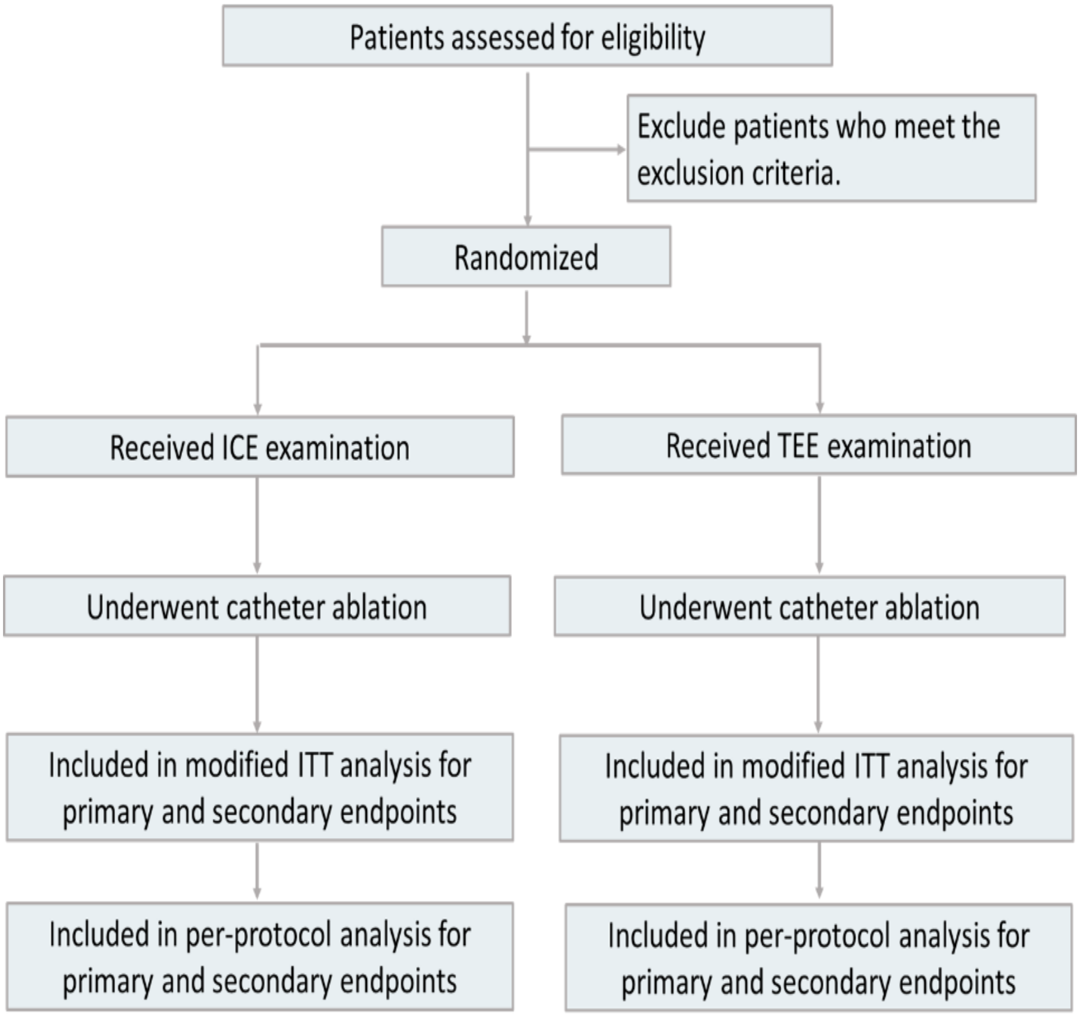

Figure 1. Trial design

3. Methods: Participants, Sample size justification , Interventions, and Endpoints

3.1 Study Setting

This multicenter study was conducted at 10 tertiary medical institutions across China, including Shanghai Chest Hospital, Ren Ji Hospital, and eight additional high-volume centers specializing in AF ablation (see Appendix for full list of participating sites). All centers possess substantial expertise in AF ablation and intracardiac imaging, thereby ensuring strict adherence to the study protocol and high procedural quality.

3.2 Sample size justification

The sample size calculation is based on the hypothesis and study design. For this non-

inferiority trial, the following parameters were used:

Type I error (α) = 0.025, corresponding to a Z-value (Z_α) of 1.96

Type II error (β) = 0.2, corresponding to a Z-value (Z_β) of 0.84

Expected event rate in the ICE group (p_1) = 0.6%

Expected event rate in the TEE group (p_2) = 0.5%

Non-inferiority margin (Δ) = 0.8%

These values were plugged into the sample size calculation formula for non-inferiority studies:

$$n = (Z_\alpha + Z_\beta)^2*(p_1*(1 - p_1) + p_2*(1 - p_2))/(p_1 - p_2 - \Delta)^2$$

$$n = (1.96 + 0.84)^2*(0.006*(1 - 0.006) + 0.005*(1 - 0.005))/(0.006 - 0.005 - 0.008)^2$$

$$n = 7.84*(0.006*0.994 + 0.005*0.995)/(-0.007)^2$$

$$n = 7.84*0.010939/0.000049$$

$$n = 1750.45$$

Based on this calculation, a sample size of approximately 1750 patients is required to achieve 80% power at a 5% significance level, assuming a non-inferiority margin of 0.8%. The actual recruited sample size of 1810 patients is slightly higher than the calculated sample size, indicating that the study's statistical power might be slightly higher than the anticipated 80%.

If the expected dropout rate is 2%, the sample size should be adjusted accordingly. The adjusted sample size can be calculated using the following formula: Adjusted sample size = Original sample size/(1 - Dropout rate)

In this case, the original sample size is 1750 patients (875 in each group). Applying the dropout rate adjustment: Adjusted sample size =1750/(1-0.02) =1750/0.98 =1786 (rounded up).

Therefore, to maintain the desired statistical power and significance level while accounting for the expected 2% dropout rate, a total of 1786 patients (approximately 893 in each group) should be recruited.

Rationale for Non-Inferiority Margin

The non-inferiority margin (Δ = 0.8%) was selected based on clinical and statistical justifications. Although the anticipated difference between the ICE and TEE groups is only 0.1%, the margin reflects the maximum clinically acceptable increase in thromboembolic risk, as supported by prior literature and expert consensus. In the context of extremely low event rates (0.5% – 0.6%), using a wider margin allows for a feasible study sample size without compromising patient safety or trial integrity. Furthermore, demonstrating that the upper limit of the 95% confidence interval remains within the 0.8% margin ensures clinical comparability. This design strategy is consistent with FDA and EMA guidance for non-inferiority trials with binary safety endpoints.

3.3 Eligibility Criteria

Participants undergoing atrial fibrillation ablation on a stable, uninterrupted regimen of oral anticoagulation for at least 3 weeks—either with a vitamin K antagonist (with INR maintained between 2.0 and 3.0) or with a direct oral anticoagulant (DOAC)—were eligible for enrollment.

Inclusion Criteria

Participants must meet all of the following criteria:

1. Aged 18 to 80 years
2. Diagnosed with paroxysmal, persistent, or long-standing persistent AF based on ECG or Holter
3. Scheduled to undergo radiofrequency catheter ablation
4. Able and willing to provide written informed consent

Exclusion Criteria

Participants will be excluded if they meet any of the following:

1. End-stage disease with a life expectancy of less than 1 year.
2. Severe heart failure, defined as New York Heart Association (NYHA) functional class III or IV, or a most recently documented left ventricular ejection fraction (LVEF) <30%.
3. History of prior AF treatment with surgical or catheter ablation.
4. Bradyarrhythmia requiring pacemaker therapy or the presence of an implanted implantable cardioverter-defibrillator (ICD).
5. Recent major cardiovascular events, including acute myocardial infarction (AMI), any percutaneous coronary intervention (PCI), cardiac valve surgery, or percutaneous valvular intervention within the past 3 months.
6. Stroke or transient ischemic attack (TIA) within the past 6 months.
7. Active systemic infection or sepsis at the time of enrollment.
8. Contraindications to TEE, including but not limited to esophageal disease (e.g., esophageal stricture, esophageal varices), recent upper gastrointestinal bleeding, or severe coagulopathy.
9. Participation in another investigational study involving devices or pharmaceutical agents within the past 30 days.
10. Women of childbearing potential who are pregnant, breastfeeding, or planning to become pregnant during the study period.

3.4 Interventions

ICE Group (Intervention Arm)

ICE imaging was performed using a 10F SoundStar probe (Biosense Webster Inc., Diamond Bar, CA), which was inserted through an 11F hemostatic sheath positioned in the left femoral vein. ICE catheters are equipped with a linear-phased array multi-frequency (5.5-10 MHz) transducer and connected to the Vivid I system (GE Healthcare, Waukesha, WI).

Procedure Steps:

1. **Positioning the ICE Catheter**:
    - The tip of the ICE catheter was placed inside the mid-right atrium and rotated clockwise until the LAA was visualized.
    - Subsequently, the ICE probe was positioned parallel to the His bundle to obtain long-axis imaging of the LAA.
    - The probe was then advanced into the right ventricular outflow tract (RVOT) and pulmonary artery (PA) to achieve better visualization of the LAA.

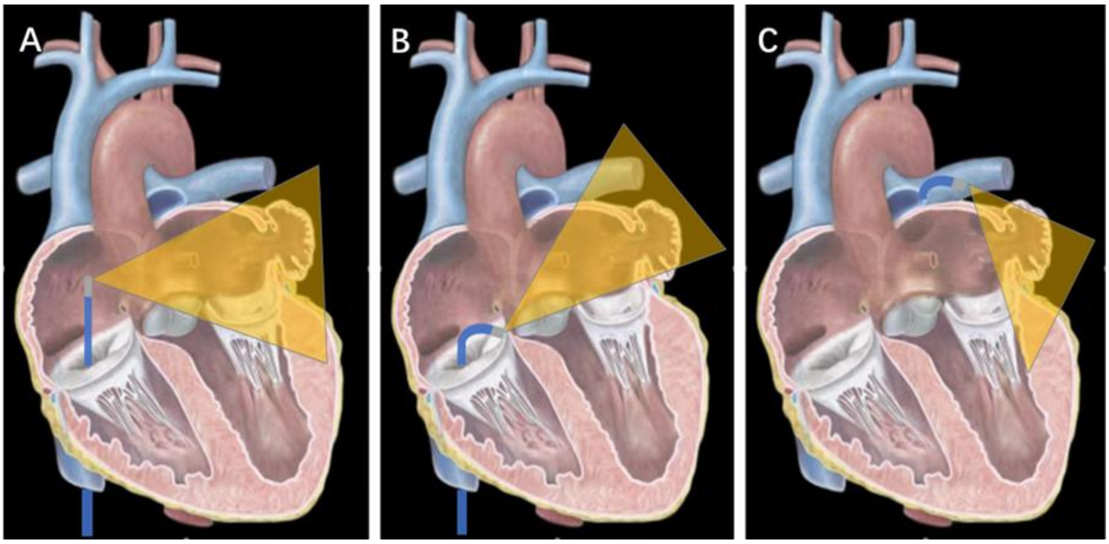

Figure 2. Detection of thrombus of LA/LAA from different positions

2. **Minimized Fluoroscopy Strategy**:
    - During procedures utilizing the ICE catheter, a strategy of minimized fluoroscopy was employed, although achieving zero radiation exposure was not mandatory.
    - Specifically, ICE catheter and CARTSOUNDTM integrated real-time ICE imaging were utilized for:
        - Geometry construction
        - Transseptal puncture
        - Effusion monitoring

- Minimal fluoroscopy was utilized at each step as determined feasible by the individual operator.

This approach ensures comprehensive imaging while minimizing patient exposure to radiation. The use of real-time ICE imaging facilitates precise procedural guidance and enhances safety during complex cardiac interventions.

TEE Group (Control Arm)

- Thrombus screening performed within 24 hours pre-procedure using standard TEE (GE Vivid E9 or Philips iE33).
- Multiplanar views including mid-esophageal four-chamber view, mid-esophageal two-chamber view, mid-esophageal long-axis view, transgastric short-axis view, deep transgastric long-axis view and upper esophageal aortic arch view.
- Transseptal puncture guided by fluoroscopy.

TEE images were first evaluated by an experienced echocardiographer and subsequently reviewed by at least two other experienced echocardiographers who were blinded to the results of the initial TEE. This meticulous procedure ensures comprehensive cardiac assessment and quality control through multiple expert reviews.

Ablation Procedure (Both Groups)

- Ablation performed using THERMOCOOL SMARTTOUCH® SurroundFlow catheter under CARTO3 guidance.
- PentaRay® mapping catheter used for 3D reconstruction.
- Ablation strategy: pulmonary vein isolation (PVI) as standard, with lesion settings: 35–50W power, AI targets: 450–550 (anterior), 350–450 (posterior), – Saline flow as per manufacturer guidelines.
- Intraprocedural activated clotting time (ACT) was monitored at regular intervals, with intravenous heparin administered to maintain an ACT between 300 and 350 seconds throughout the procedure.

3.5 Endpoints

Primary Endpoints

The primary endpoint centers on thromboembolic events occurring during the periprocedural period, which encompass:

- Stroke: Characterized as an abrupt onset of neurological dysfunction resulting from disrupted cerebral blood flow, causing tissue damage. Diagnosis is confirmed via MRI or CT imaging.
- Transient Ischemic Attack (TIA): A brief episode of neurological dysfunction due to temporary interruption of blood supply to a localized area of the brain. Symptoms typically resolve within 24 hours.
- Systemic Embolism: Sudden blockage of a blood vessel by an embolus, resulting in tissue ischemia or infarction in an organ other than the brain.

Secondary Endpoints

The prespecified secondary endpoints include:

- The detection rate of thrombus.
- Death related to the radiofrequency ablation procedure.
- Major bleeding complications: pericardial effusions and cardiac tamponade.
- Minor bleeding complications: vascular complications such as pseudoaneurysms, arterio-venous fistulas, and hematomas.
- Procedural characteristics: total procedure time, fluoroscopy time, and radiofrequency delivery time.
- Pre-procedural psychological state: Assessed using the Hospital Anxiety and Depression Scale (HADS), [14]a self-assessment questionnaire measuring anxiety and depression levels in patients receiving medical care. Higher scores indicate more severe symptoms.
- Procedural pain degree: Measured using the Numeric Rating Scale (NRS), where patients rate their pain on a scale from 0 (no pain) to 10 (worst possible pain).
- Hospitalization cost: Expenses incurred during a patient's hospital stay, including charges for medical services, procedures, medications, accommodations, and other related expenses.
- Preprocedural waiting time: The period of time a patient waits before undergoing ablation.

These well-defined endpoints will help the researchers assess the effectiveness, safety, and patient experience of using ICE compared to TEE in patients undergoing AF ablation.

4. Methods: Assignment of Interventions

4.1 Allocation

This study employs a **simple computerized randomization** strategy with a 1:1 allocation ratio. Eligible participants are randomly assigned to undergo thrombus screening with either ICE (ICE group) or TEE (TEE group) before catheter ablation.

The randomization schedule is generated using validated, centralized computer software to ensure adequate allocation concealment. Allocation occurs **after patient enrollment and informed consent**, and prior to the start of imaging and ablation procedures.

4.2 Blinding (Masking)

This study is open-label due to the nature of the interventions—ICE and TEE are procedurally distinct and cannot be blinded to operators or patients. However, to minimize bias:

- The Clinical Endpoint Committee (CEC) is blinded to group assignment when reviewing clinical outcomes related to thromboembolic and bleeding events.
- Statistical analysts will also remain blinded to group allocation during primary endpoint analysis.

No blinding is implemented for care providers or procedural staff due to the technical impossibility and potential risk to procedural safety.

4.3 Implementation

- The randomization sequence is generated centrally by the study's data coordination center.
- Site investigators are responsible for enrolling eligible participants and confirming inclusion/exclusion criteria.

- Random assignment is implemented electronically through the electronic data capture (EDC) system, which automatically provides allocation after patient registration.

All randomization actions are timestamped and logged within the EDC system to ensure traceability and compliance.

5. Methods: Data Collection, Management, Quality Control and Analysis

5.1 Data Collection Methods

All patient data are recorded using a secure electronic Case Report Form (eCRF) system. Data collection is performed by trained investigators at each participating center and includes:

Baseline Data

- Demographics: age, sex, height, weight, blood pressure
- Medical history: cardiovascular disease, non-cardiac comorbidities
- AF history: date of onset, type (paroxysmal/persistent), CHA_2DS_2-VASc score
- Medications: antiarrhythmic drugs, anticoagulants
- Echocardiographic parameters: LVEF, left atrial diameter
- ECG: heart rate, rhythm

Procedural Data

- Imaging modality used (ICE or TEE)
- Imaging views and interpretation
- Mapping system and catheter type
- Transseptal puncture method and anticoagulation strategy
- Radiofrequency power, ablation duration, lesion characteristics

Outcome and Follow-Up Data

- Thromboembolic events (stroke, TIA, systemic embolism)
- Complications: bleeding, tamponade, vascular injury
- Pain and anxiety scores (NRS and HADS)
- Hospitalization cost and duration
- Follow-up visits at 1 week, 2 weeks, and 1 month post-procedure

- Adverse events and serious adverse events (SAEs)

All collected data are entered in real time or within 14 days of each study visit, and validated by the site principal investigator.

5.2 Data Management

A central **data management team** oversees the trial-wide eCRF system. The following procedures are in place:

- **Data entry validation**: automated logic and range checks within the eCRF system
- **Source data verification**: routine site monitoring by CRO staff
- **Data security**: encrypted server storage with access limited to authorized personnel
- **Audit trail**: all modifications timestamped and traceable
- **Missing data protocol**: queries issued and resolved in collaboration with local site staff

All data will be locked prior to final analysis after resolving outstanding queries. Patient confidentiality is protected throughout by anonymization and secure data handling procedures.

5.4 Quality Control

To ensure the validity and consistency of thrombus assessment by ICE and TEE, the following quality control measures are implemented:

Standardized Imaging Protocols

- All participating sites are required to follow a unified imaging acquisition protocol for both intracardiac echocardiography (ICE) and transesophageal echocardiography (TEE), based on current international guidelines.
- For ICE, standardized views from the pulmonary artery (PA) or right ventricular outflow tract (RVOT) are mandated for optimal visualization of the left atrial appendage (LAA).
- For TEE, imaging is performed at multiple standard angles (0°, 45°, 90°, 135°) to comprehensively assess the LAA.

Operator Training and Credentialing

- All echocardiographers and electrophysiologists involved in image acquisition and interpretation received centralized training before study initiation.
- Competency was verified through practical assessment and review of sample cases to ensure proficiency with both ICE and TEE modalities.

Blinded Independent Image Interpretation

- All TEE and ICE images are independently reviewed by at least two experienced echocardiographers who are blinded to group assignment and to each other's assessments.
- In cases of disagreement, a third independent reviewer adjudicates the final interpretation.
- Image reviewers are unaware of patients' clinical outcomes to prevent interpretation bias.

5.4 Statistical Methods

Primary Analysis Population

- Modified intention-to-treat (mITT): all randomized patients who underwent ablation
- Per-protocol (PP): patients who completed imaging and ablation per assigned group without major protocol violations

Primary Endpoint Analysis

- Incidence of thromboembolic events will be compared between ICE and TEE groups.
- Analysis will use logistic regression to estimate the risk difference and 95% confidence interval.
- Non-inferiority is demonstrated if the upper bound of the 95% CI for the risk difference is below the predefined margin of 0.8%.
- The primary endpoint was evaluated using risk difference (RD) with Farrington – Manning 95% CI, and noninferiority was concluded if the upper CI limit was below the prespecified margin.

Secondary Endpoint Analysis

- Continuous variables (e.g., procedure time, pain scores): Presented as mean ± SD or median (IQR); Compared using t-test or Wilcoxon rank-sum test (based on normality).
- Categorical variables (e.g., thrombus detection, bleeding): Expressed as counts and percentages and compared using chi-square or Fisher's exact test.
- Binary secondary endpoints were reported as relative risk (RR) with 95% CIs; continuous endpoints were expressed as mean difference (MD) with 95% CIs.
-

Missing Data

- Primary endpoint: patients lost to follow-up before event occurrence will be excluded from the per-protocol analysis
- Sensitivity analyses with imputation may be performed as needed

Subgroup and Sensitivity Analyses

- Predefined subgroups: age, sex, AF type, CHA_2DS_2-VASc score, imaging quality
- Sensitivity: excluding equivocal imaging cases, or based on procedural adherence

All analyses will be conducted using SAS (v9.4).

6. Methods: Monitoring

6.1 Data Monitoring

An independent Data Safety Monitoring Board (DSMB) has been established for this study to ensure patient safety and maintain the scientific integrity of the trial. The DSMB operates independently from the study sponsor and investigators, in accordance with a prespecified DSMB charter.

In addition to the DSMB, the following oversight mechanisms are in place:

Steering Committee:

- Provides overall scientific and strategic oversight of the study.
- Reviews protocol deviations and serious adverse events (SAEs).
- Supervises enrollment progress and data quality across participating centers.

Clinical Endpoint Committee (CEC):

- An independent, blinded committee composed of experts in electrophysiology and neurology.
- Responsible for adjudicating all suspected thromboembolic events and major complications.
- Operates under a prespecified charter, with access only to de-identified clinical event summaries and relevant imaging.
- These monitoring structures ensure rigorous, impartial assessment of safety and efficacy outcomes throughout the trial.

6.2 Auditing

Monitoring of the clinical trial is coordinated by the CRO designated by the sponsor. The CRO conducts routine audits and site monitoring visits based on a risk-based monitoring plan.

Key Monitoring Activities Include:

- Source data verification (SDV): Ensuring accuracy and completeness of entries in the eCRF relative to source medical records
- Protocol compliance checks: Evaluation of adherence to inclusion/exclusion criteria, procedural protocol, and follow-up schedule
- Informed consent review: Verification of documentation and regulatory compliance
- Adverse event reporting: Ensuring all AEs and SAEs are appropriately documented and reported within required timelines

Monitoring frequency is adapted based on site performance, recruitment rate, and data quality indicators. Critical and major findings are addressed promptly in collaboration with the site's principal investigator and sponsor.

7. Ethics and Dissemination

7.1 Research Ethics Approval

This study is conducted in full compliance with the Declaration of Helsinki, ISO 14155, and all applicable national and local regulatory requirements governing human research.

Prior to initiation at each participating center, the complete clinical investigational plan—including the protocol, patient information sheet, and informed consent form—was submitted to and approved by the Institutional Review Board (IRB) / Ethics Committee (EC) of that center.

Any protocol amendments, including changes to eligibility criteria, outcomes, or study procedures, will be re-submitted to the EC/IRB for review and approval prior to implementation. Serious Adverse Events (SAEs) and Unexpected Adverse Device Effects (UADEs) will be reported to the EC in accordance with institutional and regulatory timelines.

7.2 Protocol Amendments

All significant changes to the protocol will be documented in a version-controlled amendment log, specifying the rationale for modification, date of implementation, and impact on study conduct. Updates will be communicated to:

- Ethics Committees / IRBs
- Participating investigators and research sites

7.3 Informed Consent

All participants or their legally authorized representatives must voluntarily sign a written informed consent form before undergoing any study-specific procedure. The consent process ensures that participants are adequately informed of:

- The study objectives and procedures
- Potential benefits and risks
- Data confidentiality protection
- Voluntary participation and right to withdraw at any time

The process is documented in accordance with Good Clinical Practice (GCP) and ISO 14155 standards.

7.4 Confidentiality

Participant confidentiality is strictly protected throughout the study:

- All data are pseudonymized using unique study identifiers
- Personal identifiers are stored separately from clinical data
- Access to identifiable information is restricted to authorized research personnel
- Electronic data is stored in password-protected and encrypted systems

Data handling complies with all relevant data protection laws in China and institutional policies.

7.5 Declaration of Interests

Dr. Xu Liu, the principal investigator of this study, has received grant support from Biosense Webster Inc. (USA). This funding is unrelated to the current trial and does not influence the study design, conduct, or interpretation.

7.6 Access to Data

The sponsor and principal investigators will have full access to the final de-identified dataset. Requests for data access by external researchers will be considered upon reasonable request, subject to ethical approval and data-sharing agreements.

7.7 Ancillary and Post-Trial Care

Study-related procedures are covered by standard clinical care. No study-specific insurance is provided. Any trial-related adverse events requiring medical care will be managed by the treating institution in accordance with hospital policies and national clinical practice guidelines.

8. Protocol modifications

This clinical study underwent the following modifications during its course:

8.1 Eligibility Criteria Modification

Two key changes were made to the exclusion criteria in the updated protocol:

1. Removal of uncontrolled hypertension: The previous exclusion of patients with uncontrolled hypertension was eliminated, as this condition is common in the AF

population and can be managed during the study period.
2. Addition of TEE contraindications: A new exclusion criterion was added for patients with contraindications to transesophageal echocardiography (TEE), including esophageal disease, recent upper gastrointestinal bleeding, or severe coagulopathy, to ensure procedural safety.

8.2 Primary Study Endpoints Modification

The primary study endpoints were adjusted from encompassing all complications to focus specifically on peri-procedural thromboembolic events (stroke, transient ischemic attack, and systemic embolism). Other non-thromboembolic complications (bleeding complications) were included as secondary study endpoints.

8.3 Planned Sample Size Adjustment

The originally planned sample size of 1200 cases was increased to 1786 due to modification in the primary study endpoints during the trial. Detailed sample size calculation methods are outlined in Section 3.2.

8.4 Additional Secondary Endpoints

Secondary endpoints were expanded to include assessments such as pre-procedural anxiety and depression states, procedural pain level, post-procedural pain or discomfort, hospitalization costs, and pre-procedural waiting time.

8.5 Study Centers Change

Eight centers in mainland China initially participated in this randomized trial as following:
1.Shanghai Chest Hospital Shanghai, China
2.Sir Run Run Shaw Hospital
3.Beijing Chao Yang Hospital
4.First Affiliated Hospital of Suzhou Medical College
5.Qilu Hospital of Shandong University
6.RenJi Hospital
7.The First Affiliated Hospital with Nanjing Medical University
8.Qianfoshan Hospital
Due to the COVID-19 pandemic and strict local containment policies, enrollment progress in some centers was slow and stagnant in China. We adjusted several centers, and in August 2022, they participated in the multicenter randomized controlled trial. Hence, a total of 10 centers participated in this randomized trial.
1. Shanghai Chest Hospital, Shanghai, China

2. Ren Ji Hospital, Shanghai Jiao Tong University School of Medicine, Shanghai, China

3.The Central Hospital of Wuhan, Tongji Medical College, Huazhong University of Science and Technology, Wuhan, China

4.Affiliated Hospital of Jining Medical University, Jining, China

5.Second Affiliated Hospital of Shandong University of Traditional Chinese Medicine, Jinan, China

6.Yuhuan Second People's Hospital, Yuhuan, China

7.Changshu Hospital of Traditional Chinese Medicine, Changshu, China

8.The PLA Navy Anqing Hospital, Anqing, China

9.Jinan City People’s Hospital, Jinan, China

10.Xuzhou Central Hospital, Xuzhou, China

9. Appendices

9.1 Appendix A: List of Abbreviations

Abbreviation	Full Term
ACC	American College of Cardiology
ACT	Activated Clotting Time
ADE	Adverse Device Effect
AE	Adverse Event
AF	Atrial Fibrillation
AHA	American Heart Association
AI	Ablation Index
AMI	Acute Myocardial Infarction
APHRS	Asia Pacific Heart Rhythm Society
AT	Atrial Tachycardia
CEC	Clinical Endpoint Committee
CHA_2DS_2-VASc	Congestive heart failure, Hypertension, Age ≥75, Diabetes, Stroke/TIA, Vascular disease, Age 65–74, Sex category
COVID-19	Coronavirus Disease 2019
CRF	Case Report Form
CRO	Contract Research Organization
DSMB	Data Safety Monitoring Board
EC	Ethics Committee
ECG	Electrocardiogram
EDC	Electronic Data Capture
EGM	Intracardiac Electrogram
EHRA	European Heart Rhythm Association
EMA	European Medicines Agency
FDA	Food and Drug Administration
FU	Follow-Up
GCP	Good Clinical Practice
GE	General Electric

HADS	Hospital Anxiety and Depression Scale
HRS	Heart Rhythm Society
ICD	Implantable Cardioverter-Defibrillator
ICE	Intracardiac Echocardiography
INR	International Normalized Ratio
IQR	Interquartile Range
IRB	Institutional Review Board
ISO	International Organization for Standardization
LA	Left Atrium
LAA	Left Atrial Appendage
LAHRS	Latin American Heart Rhythm Society
LVEF	Left Ventricular Ejection Fraction
MRI	Magnetic Resonance Imaging
NRS	Numeric Rating Scale
NYHA	New York Heart Association
PA	Pulmonary Artery
PCI	Percutaneous Coronary Intervention
PP	Per Protocol
PVI	Pulmonary Vein Isolation
RA	Right Atrium
RVOT	Right Ventricular Outflow Tract
SAE	Serious Adverse Event
SAS	Statistical Analysis System
SD	Standard Deviation
SDV	Source Data Verification
TEE	Transesophageal Echocardiography
TIA	Transient Ischemic Attack
UADE	Unexpected Adverse Device Effect
WF	Wavefront
eCRF	Electronic Case Report Form

9. 2 Appendix B: References

1. Lurie A, Wang J, Hinnegan KJ, McIntyre WF, Belley -Côté Emilie P., Amit G, *et al.* Prevalence of Left Atrial Thrombus in Anticoagulated Patients With Atrial Fibrillation. *Journal of the American College of Cardiology* American College of Cardiology Foundation; 2021;**77**:2875–86.

2. January CT, Wann LS, Alpert JS, Calkins H, Cigarroa JE, Cleveland JC, *et al.* 2014 AHA/ACC/HRS guideline for the management of patients with atrial fibrillation: a report of the American College of Cardiology/American Heart Association Task Force on practice guidelines and the Heart Rhythm Society. *Circulation* 2014;**130**:e199-267.

3. Baran J, Stec S, Pilichowska-Paszkiet E, Zaborska B, Sikora-Frąc M, Kryński T, *et al.* Intracardiac echocardiography for detection of thrombus in the left atrial appendage: comparison with transesophageal echocardiography in patients undergoing ablation for atrial fibrillation: the Action-Ice I Study. *Circulation Arrhythmia and Electrophysiology*

2013;**6**:1074–81.

4. Baran J, Zaborska B, Piotrowski R, Sikora-Frąc M, Pilichowska-Paszkiet E, Kułakowski P. Intracardiac echocardiography for verification for left atrial appendage thrombus presence detected by transesophageal echocardiography: the ActionICE II study. *Clin Cardiol* 2017;**40**:450.

5. January CT, Wann LS, Calkins H, Chen LY, Cigarroa JE, Cleveland JC, *et al.* 2019 AHA/ACC/HRS Focused Update of the 2014 AHA/ACC/HRS Guideline for the Management of Patients With Atrial Fibrillation: A Report of the American College of Cardiology/American Heart Association Task Force on Clinical Practice Guidelines and the Heart Rhythm Society. *J Am Coll Cardiol* 2019;**74**:104–32.

6. Hilberath JN, Oakes DA, Shernan SK, Bulwer BE, D'Ambra MN, Eltzschig HK. Safety of transesophageal echocardiography. *Journal of the American Society of Echocardiography: Official Publication of the American Society of Echocardiography* 2010;**23**:1115–27; quiz 1220–1.

7. Sriram CS, Banchs JE, Moukabary T, Moradkhan R, Gonzalez MD. Detection of left atrial thrombus by intracardiac echocardiography in patients undergoing ablation of atrial fibrillation. *J Interv Card Electrophysiol* 2015;**43**:227–36.

8. Biase LD, Briceno DF, Trivedi C, Mohanty S, Gianni C, Burkhardt JD, *et al.* Is transesophageal echocardiogram mandatory in patients undergoing ablation of atrial fibrillation with uninterrupted novel oral anticoagulants? Results from a prospective multicenter registry. *Heart Rhythm* 2016;**13**:1197–202.

9. Patel K, Natale A, Yang R, Trivedi C, Romero J, Briceno D, *et al.* Is transesophageal echocardiography necessary in patients undergoing ablation of atrial fibrillation on an uninterrupted direct oral anticoagulant regimen? Results from a prospective multicenter registry. *Heart Rhythm* 2020;**17**:2093–9.

10. Wang Y, Zhao Y, Zhou K, Zei PC, Wang Y, Cheng H, *et al.* Intracardiac echocardiography is a safe and effective alternative to transesophageal echocardiography for left atrial appendage thrombus evaluation at the time of atrial fibrillation ablation: The ICE-TEE study. *Pacing Clin Electrophysiol* 2023;**46**:3–10.

11. Saksena S, Sra J, Jordaens L, Kusumoto F, Knight B, Natale A, *et al.* A prospective comparison of cardiac imaging using intracardiac echocardiography with transesophageal echocardiography in patients with atrial fibrillation: the intracardiac echocardiography guided cardioversion helps interventional procedures study. *Circulation Arrhythmia and Electrophysiology* 2010;**3**:571–7.

12. Jingquan Z, Deyong L, Huimin C, Hua F, Xuebin H, Chenyang J, *et al.* Intracardiac echocardiography Chinese expert consensus. *Front Cardiovasc Med* Frontiers; 2022;**9**.

13. Tzeis S, Gerstenfeld EP, Kalman J, Saad E, Shamloo AS, Andrade JG, *et al.* European Heart Rhythm Association (EHRA)/Heart Rhythm Society (HRS)/Asia Pacific Heart Rhythm Society (APHRS)/Latin American Heart Rhythm Society (LAHRS) expert consensus statement on catheter and surgical ablation of atrial fibrillation. *Heart Rhythm* 2024;S1547527124002613.

14. Zigmond AS, Snaith RP. The hospital anxiety and depression scale. *Acta Psychiatr Scand* 1983;**67**:361–70.
