# Supplementary material for: Intracardiac vs Transesophageal Echocardiography in Atrial Fibrillation Ablation: A Randomized Clinical Trial
Source: JAMA Cardiol. 2025 Oct 8;10(12):1249–56. doi: 10.1001/jamacardio.2025.3687 (PMC12509080; doi:10.1001/jamacardio.2025.3687)
Supplement: Supplement 2. — eTable 1. Baseline Characteristics of Study Patients (Modified Intention-to-Treat) eTable 2. Baseline Characteristics of Study Patients (Per-Protocol Set) eTable 3. Comparison of Ablation Strategies Between the ICE and TEE Groups eTable 4. Primary End Points (Modified Intention-to-Treat) eTable 5. Primary End Points (Per-Protocol Set) eTable 6. Baseline Characteristics of Patients With Stroke or Transient Ischemic Attack eTable 7. Secondary End Points (Modified Intention-to-Treat) eTable 8. Secondary End Points (Per-Protocol Set) eTable 9. Primary End Point Subgroup by ITT Analysis eFigure 1. Diffusion-Weighted MRI Images Showing Acute Ischemic Stroke Following AF Ablation in an ICE Group Patient eFigure 2. Representative ICE and TEE Images of the Left Atrial Appendage From Different Acoustic Windows eFigure 3. Representative Images of Non–Left Atrial Appendage Thrombi Identified by ICE [file jamacardiol-e253687-s002.pdf]

Supplemental Online Content

Hu X, Jiang W, Wang X, et al; ICE vs TEE Study Investigators. Intracardiac vs transesophageal echocardiography in atrial fibrillation ablation: a randomized clinical trial. *JAMA Cardiol*. Published online October 8, 2025. doi:10.1001/jamacardio.2025.3687

eTable 1. Baseline Characteristics of Study Patients (Modified Intention-to-Treat)
eTable 2. Baseline Characteristics of Study Patients (Per-Protocol Set)
eTable 3. Comparison of Ablation Strategies Between the ICE and TEE Groups
eTable 4. Primary End Points (Modified Intention-to-Treat)
eTable 5. Primary End Points (Per-Protocol Set)
eTable 6. Baseline Characteristics of Patients With Stroke or Transient Ischemic Attack
eTable 7. Secondary End Points (Modified Intention-to-Treat)
eTable 8. Secondary End Points (Per-Protocol Set)
eTable 9. Primary End Point Subgroup by ITT Analysis
eFigure 1. Diffusion-Weighted MRI Images Showing Acute Ischemic Stroke Following AF Ablation in an ICE Group Patient
eFigure 2. Representative ICE and TEE Images of the Left Atrial Appendage From Different Acoustic Windows
eFigure 3. Representative Images of Non–Left Atrial Appendage Thrombi Identified by ICE

This supplemental material has been provided by the authors to give readers additional information about their work.

eTable 1. Baseline Characteristics of Study Patients (Modified Intention-to-Treat)

	No. (%)		P value
Characteristics	ICE group (n=888)	TEE group (n=890)	
Age, mean (SD), y	64.4 (9.4)	64.2 (9.6)	.65
Sex			
Female	421 (47.3)	433 (48.7)	.47
Male	467 (52.7)	457 (51.3)	
BMI, mean (SD)[a]	23.0 (2.1)	23.0 (2.1)	.69
Smoking status	239 (26.9)	257 (28.9)	.83
Drinking	258 (29.1)	273 (30.7)	.46
AF type			.26
Paroxysmal AF	459 (51.7)	428 (48.1)	
Persistent AF	301 (33.9)	315 (35.4)	
Long-standing AF	128 (14.4)	147 (16.5)	
CHA_2DS_2-VaSc score, mean (SD)	2.2 (1.4)	2.1 (1.4)	.71
Concomitant disease			
Hypertension	536 (60.4)	532 (59.8)	.80
Prior stroke or TIA	91 (10.3)	89 (10.0)	.86
Coronary artery disease	199 (22.4)	178 (20.0)	.21
Diabetes mellitus	139 (15.7)	149 (16.7)	.53
Heart failure	31 (3.5)	24 (2.7)	.33
Echocardiographic findings			
LAD, mean (SD), mm	43.3 (4.8)	43.1 (4.9)	.39
LVEF, mean (SD), %	62.8 (3.7)	62.6 (4.3)	.48
Anticoagulant regimen			.38
Rivaroxaban	846 (95.3)	837 (94.0)	
Dabigatran	35 (3.8)	46 (5.2)	
Vitamin K antagonist	8 (0.9)	7 (0.8)	

ICE: intracardiac echocardiography; TEE: transesophageal echocardiography; BMI: body mass index; AF: atrial fibrillation; TIA: transient ischemic attack; LAD: left atrial diameter; LVEF: left ventricle ejection fraction.

[a]Calculated as weight in kilograms divided by height in meters squared.

eTable 2. Baseline Characteristics of Study Patients (Per-Protocol Set)

	No. (%)		*P* value
Characteristics	ICE group (n = 905)	TEE group (n = 873)	
Age, mean (SD), y	64.4 (9.5)	64.2 (9.4)	.70
Sex			.73
Female	431 (47.6)	423 (48.5)	
Male	474 (52.4)	450 (51.5)	
BMI, mean (SD)[a]	23.1 (2.1)	23.0 (2.1)	.64
Smoking status	242 (26.7)	254 (29.1)	.27
Drinking	263 (29.1)	268 (30.7)	.45
AF type			.35
Paroxysmal AF	465 (51.4)	422 (47.3)	
Persistent AF	309 (34.1)	307 (35.2)	
Long-standing AF	131 (14.5)	144 (16.5)	
CHA_2DS_2-VaSc score, mean (SD)	2.2 (1.4)	2.1 (1.3)	.53
Concomitant disease			
Hypertension	547 (60.4)	521 (59.7)	.74
Prior stroke or TIA	94 (10.4)	86 (9.9)	.71
Coronary artery disease	205 (22.7)	172 (19.7)	.13
Diabetes mellitus	140 (15.5)	148 (17.0)	.40
Heart failure	32 (3.5)	23 (2.6)	.27
Echocardiographic findings			
LAD, mean (SD), mm	43.1 (4.9)	43.3 (4.8)	.43
LVEF, mean (SD), %	62.6 (4.3)	62.8 (3.7)	.31
Anticoagulant regimen			.69
Rivaroxaban	860 (95.0)	823 (94.0	
Dabigatran	37 (4.1)	43 (4.9)	
Vitamin K antagonist	8 (0.9)	7 (0.8)	

ICE: intracardiac echocardiography; TEE: transesophageal echocardiography; BMI: body mass index; AF: atrial fibrillation; TIA: transient ischemic attack; LAD: left atrial diameter; LVEF: left ventricle ejection fraction.

[a]Calculated as weight in kilograms divided by height in meters squared.

eTable 3. Comparison of Ablation Strategies Between the ICE and TEE Groups

		No. (%)		
AF type	Ablation Strategy	ICE Group (n=459)	TEE Group (n=428)	*P* values
Paroxysmal AF	Pulmonary vein isolation	459 (100)	428（100）	1
	Posterior wall isolation	0	0	1
	Mitral isthmus	0	0	NA
	Cavotricuspid isthmus	20(4.4)	17(4.0)	.77
	Superior vena cava	6(1.3)	8(1.9)	.50
Persistent AF		ICE (n=301)	TEE (n=315)	NA
	Pulmonary vein isolation	301(100)	315(100)	1
	Posterior wall isolation	99(32.9)	98(31.1)	.64
	Mitral isthmus	235(78.1)	251(79.7)	.63
	Cavotricuspid isthmus	13(4.3)	11(3.5)	.60
	Superior vena cava	0	0	NA
Longstanding Persistent AF		ICE (n=128)	TEE (n=147)	
	Pulmonary vein isolation	128(100)	147(100)	1
	Posterior wall isolation	56(43.8)	60(40.8)	.62
	Mitral isthmus	111(86.7)	123(83.7)	.48
	Cavotricuspid isthmus	0	0	NA
	Superior vena cava	0	0	NA

AF: atrial fibrillation; ICE: intracardiac echocardiography; TEE: transesophageal echocardiography; NA = not applicable.

eTable 4. Primary End Points (Modified Intention-to-Treat)

	No. (%)			
Primary endpoints	ICE group n=888	TEE group n=890	Treatment effect* (95% CI)	*P* value
Thromboembolic events	4(0.4)	5(0.6)	RD -0.11% (-0.91% to 0.66%)	P for noninferiority = .01
Stroke	1(0.1)	0(0)		
TIA	3(0.3)	5(0.6)		
Systemic embolism	0(0)	0(0)		

ICE: intracardiac echocardiography; TEE: transesophageal echocardiography; TIA: transient ischemic attack.

*Treatment effect is presented as risk difference (RD) with 95% confidence intervals for binary outcomes.

eTable 5. Primary End Points (Per-Protocol Set)

	No. (%)			
Primary endpoints	ICE group n=905	TEE group n=873	Treatment effect* (95% CI)	P value
Thromboembolic events	4(0.4)	5(0.6)	-0.13% (-0.79% to 0.53%)	P for noninferiority = .02
Stroke	1(0.1)	0(0)		
TIA	3(0.3)	5(0.6)		
Systemic embolism	0(0)	0(0)		

ICE: intracardiac echocardiography; TEE: transesophageal echocardiography; TIA: transient ischemic attack.

*Treatment effect is presented as risk difference (RD) with 95% confidence intervals for binary outcomes.

eTable 6. Baseline Characteristics of Patients With Stroke or Transient Ischemic Attack

Patient	Group	Stroke/TIA	Age	Sex	AF type	Rhythm at event	OAC use at event	Comorbidities	CHA2DS2-VASc Score	History of stroke
1	ICE	TIA	74	Female	PAF	SR	Yes	HBP, CAD	4	No
2	ICE	TIA	68	Female	PAF	SR	Yes	CAD, DM	4	No
3	ICE	Stroke	65	Male	PerAF	SR	Yes	HBP, DM	3	No
4	ICE	TIA	76	Female	PerAF	SR	Yes	HBP	4	No
5	TEE	TIA	79	Male	LS-PerAF	AF	Yes	None	2	No
6	TEE	TIA	74	Female	PAF	SR	Yes	None	2	No
7	TEE	TIA	60	Male	PerAF	SR	Yes	HBP, CAD, DM	3	No
8	TEE	TIA	60	Male	LS-PerAF	SR	Yes	HBP, DM	2	No
9	TEE	TIA	79	Male	LS-PerAF	AF	Yes	None	2	No

AF, atrial fibrillation; CAD, coronary artery disease; DM, diabetes mellitus; HBP, hypertension; ICE, intracardiac echocardiography; TEE, transesophageal echocardiography; LS-PerAF, long-standing persistent atrial fibrillation; OAC, oral anticoagulation; PAF, paroxysmal atrial fibrillation; PerAF, persistent atrial fibrillation; SR, sinus rhythm; TIA, transient ischemic attack.

eTable 7. Secondary End Points (Modified Intention-to-Treat)

	No. (%)			
Secondary endpoints	ICE group n=888	TEE group n=890	Treatment effect, relative risk (95% CI)	P value
Presence of thrombus,	-	-		
LAA	-	-		
Non-LAA	-	-		
Death	0	0		
Major bleeding events	7(0.8)	16(1.8)	0.44 (0.18–1.06)	0.060
Pericardial effusions	5(0.6)	7(0.8)		
Cardiac tamponade requiring pericardiocentesis or surgery	2(0.2)	9(1.0)		
Major bleeding related with transseptal puncture	2(0.2)	11(1.2)	0.18 (0.04–0.82)	0.012
Minor bleeding events	10(1.1)	10(1.1)	1.00 (0.42–2.40)	0.99
Groin hematoma	8(0.9)	7(0.8)		
Pseudoaneurysm	2(0.2)	3(0.3)		
Arterio-venous fistulas	0	0		
Total procedure time, mean (SD), min	159.6±25.4	144.9±24.8	14.7 (12.37 to 17.03)	<.001
Radiofrequency delivery time, mean (SD), min	56.4±10.7	56.0±11.7	0.40 (–0.64 to 1.44)	.45
Fluoroscopy time, mean (SD), min	4.2±1.5	9.3±3.0	-5.10 (-5.32 to -4.88)	<.001
Preprocedural waiting time, mean (SD), min	14.4±8.0	23.7±10.6	-9.30 (-10.17 to -8.43)	<.001
Preprocedural anxiety and depressive state	219(24.7)	329(37)	0.67(0.57–0.78)	<.001
Procedural pain (NRS11 score, median [IQR])	0(0,0)	2(1,3)	NA	<.001

ICE, intracardiac echocardiography; TEE, transesophageal echocardiography; LAA: left atrial appendage; HADS, Hospital Anxiety and Depression Scale; SD, standard deviation; RR, relative risk;

CI, confidence interval; NRS11, Numeric Rating Scale 11; IQR, interquartile range; NA, not applicable

*Treatment effect is presented as mean difference (MD) for continuous outcomes, relative risk (RR) for binary outcomes.

eTable 8. Secondary End Points (Per-Protocol Set)

	No. (%)			
Secondary endpoints	ICE group n=905	TEE group n=873	Treatment effect* (95% CI)	P value
Presence of thrombus,	-	-	-	-
LAA	-	-	-	-
Non-LAA	-	-	-	-
Death	0	0		1
Major bleeding events	8(0.9)	15(1.7)	0.51 (0.22–1.21)	.14
Pericardial effusions	5(0.6)	7(0.8)		
Cardiac tamponade requiring pericardiocentesis or surgery	3(0.3)	8(0.9)		
Major bleeding related with transseptal puncture	3(0.3)	10(1.2)	0.29 (0.08–1.05)	.05
Minor bleeding events	11(1.2)	9(1.0)	1.18 (0.49–2.83)	.82
Groin hematoma	8(0.9)	7(0.8)		
Pseudoaneurysm	3(0.3)	2(0.2)		
Arterio-venous fistulas	0	0		
Total procedure time, mean (SD), min	159.4±25.5	144.7±24.8	14.7 (12.36 to 17.04)	<.001
Radiofrequency delivery time, mean (SD), min	56.4±10.7	56.0±11.7	0.40 (–0.64 to 1.44)	.44
Fluoroscopy time, mean (SD), min	4.3±1.6	9.4±3.0	-5.10 (-5.32 to -4.88)	<.001
Preprocedural waiting time, mean (SD), min	14.4±8.0	23.5±10.6	-9.10 (-9.98 to -8.22)	<.001
Preprocedural anxiety and depressive state	226(25.0)	322(36.9)	0.68 (0.59–0.79)	<.001
Procedural pain (NRS11 score, median [IQR])	0(0,0)	2(1,3)	NA	<.001

ICE, intracardiac echocardiography; TEE, transesophageal echocardiography; LAA: left atrial appendage; HADS, Hospital Anxiety and Depression Scale; SD, standard deviation; RR, relative risk; CI, confidence interval; NRS11, Numeric Rating Scale 11; IQR, interquartile range; NA, not applicable

*Treatment effect is presented as mean difference (MD) for continuous outcomes, relative risk (RR) for binary outcomes.

eTable 9. Primary End Point Subgroup by ITT Analysis

	No. of events			
Subgroup	ICE	TEE	Treatment effect* (95% CI)	P value
Age, y				
<65	2/414	1/422	0.49(0.04-5.42)	.56
≥65	3/490	3/484	1.01(0.20-5.04)	.99
Sex				
Male	2/464	1/478	0.48(0.04-5.36)	.55
Female	3/440	3/428	1.03(0.21-5.12)	.97
BMI, kg/m^2				
<22	2/280	2/271	1.03(0.15-7.39)	.97
≥22	3/624	2/635	0.65(0.11-3.93)	.64
AF type				
Paroxysmal	1/428	1/459	0.93(0.06-14.95)	.96
Non-paroxysmal	4/476	2/447	0.79(0.18-3.58)	.77
CHA2DS2-VASc score				
<3	1/569	1/575	0.99(0.06-15.59)	.99
≥3	4/335	3/331	0.76(0.17-3.41)	.72
LAD, mm				
<45	1/554	1/585	0.95(0.06-15.18)	.97
≥45	4/350	3/321	0.82(0.18-3.67)	.79

ICE, intracardiac echocardiography; TEE, transesophageal echocardiography; BMI: body mass index; AF: atrial fibrillation; LAD: left atrial diameter

*Treatment effect is presented relative risk (RR) for binary outcomes.

eFigure 1. Diffusion-Weighted MRI Images Showing Acute Ischemic Stroke Following AF Ablation in an ICE Group Patient

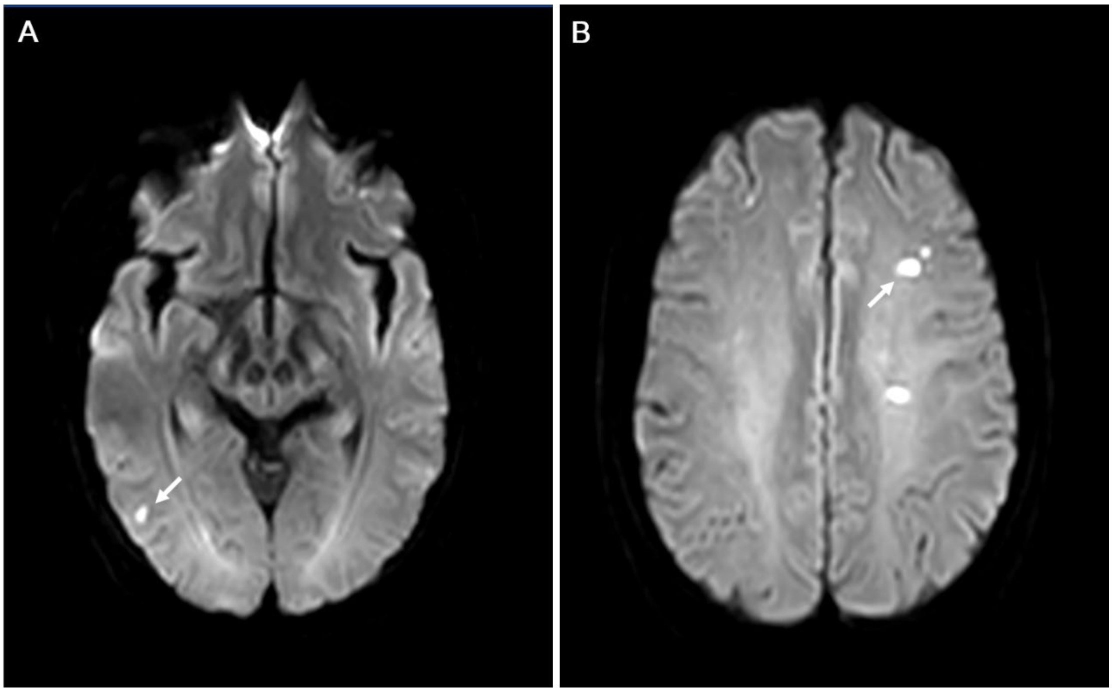

Representative diffusion-weighted MRI images from a patient in the intracardiac echocardiography (ICE) group who developed an acute ischemic stroke one day after catheter ablation for persistent atrial fibrillation. The patient had a CHA_2DS_2-VASc score of 3 and underwent pulmonary vein isolation and driver ablation, with sinus rhythm restored by direct current cardioversion. Left panel, Acute infarction in the right occipital lobe. Right panel, Acute infarction in the left frontal lobe.

White arrows indicate areas of restricted diffusion consistent with acute ischemia.

eFigure 2. Representative ICE and TEE Images of the Left Atrial Appendage From Different Acoustic Windows

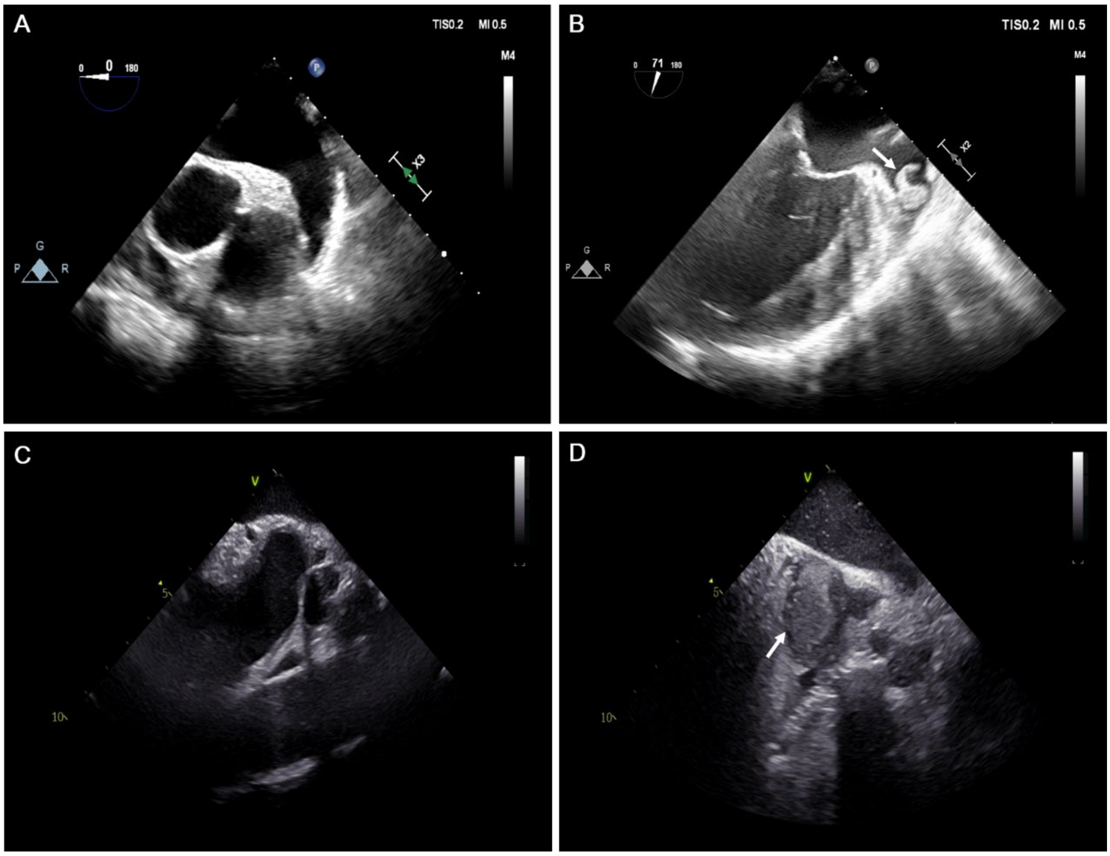

Four representative images of the left atrial appendage (LAA) acquired by intracardiac echocardiography (ICE) and transesophageal echocardiography (TEE) from different anatomical views.

A, ICE view from the pulmonary artery showing an LAA without thrombus.

B, ICE view from the pulmonary artery revealing an LAA thrombus.

C, TEE view from the posterior wall of the left atrium showing an LAA without thrombus.

D, TEE view from the posterior wall of the left atrium detecting an LAA thrombus.

White arrows denote the location of thrombi in panels B and D.

eFigure 3. Representative Images of Non–Left Atrial Appendage Thrombi Identified by ICE

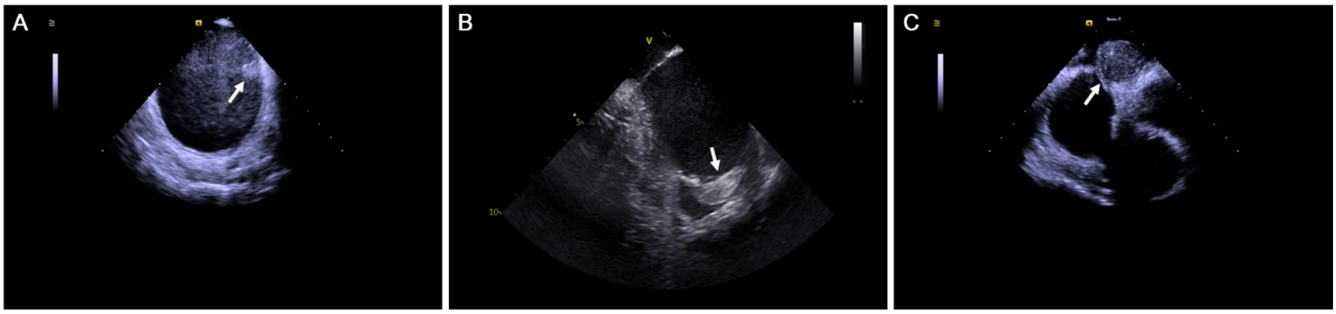

Three representative thrombi at non – left atrial appendage (non-LAA) sites visualized using intracardiac echocardiography (ICE) from the right atrium.
A, Thrombus adherent to the roof of the left atrium.
B, Spherical thrombus attached to the interatrial septal surface.
C, Thrombus located at the left pulmonary vein ridge.
White arrows indicate the location of each thrombus.
