# Supplementary material for: Intracardiac vs Transesophageal Echocardiography in Atrial Fibrillation Ablation: A Randomized Clinical Trial
Source: JAMA Cardiol. 2025 Oct 8;10(12):1249–56. doi: 10.1001/jamacardio.2025.3687 (PMC12509080; doi:10.1001/jamacardio.2025.3687)
Supplement: Supplement 3. — ICE vs TEE Study Investigators [file jamacardiol-e253687-s003.pdf]

Supplemental Online Content: Nonauthor Collaborators
*First name, last name, and suffix (if applicable) are required and will appear in PubMed.

*Group Name(s): ICE vs TEE Study Investigators							
***First Name and Middle Initial(s)**	***Last Name**	***Suffix (eg, Jr, III)**	Academic Degrees	Institution	Location (city, state/province, country)	Role or Contribution, eg, chair, principal investigator	Group (if more than 1 Group listed in the byline) and/or Subgroup (eg, Steering Committee)
Li	Zhou		MD	Shanghai Chest Hospital,Shanghai Jiao Tong University School of Medicine	Shanghai, China	Patient recruitment	
Yang	Liu		MD	Shanghai Chest Hospital,Shanghai Jiao Tong University School of Medicine	Shanghai, China	Patient recruitment	
Shaohui	Wu		MD	Shanghai Chest Hospital,Shanghai Jiao Tong University School of Medicine	Shanghai, China	Patient recruitment	
Kai	Xu		MD	Shanghai Chest Hospital,Shanghai Jiao Tong University School of Medicine	Shanghai, China	Patient recruitment	
Kaige	Li		MD	Shanghai Chest Hospital,Shanghai Jiao Tong University School of Medicine	Shanghai, China	Patient recruitment	
Ziliang	Song		MD	Shanghai Chest Hospital,Shanghai Jiao Tong University School of Medicine	Shanghai, China	Patient recruitment	
Lingcong	Kong		MD	Ren Ji Hospital, Shanghai Jiao Tong University Shanghai School of Medicine,	Shanghai, China	Patient recruitment	
Jiamin	Niu		MD	Jinan People’s Hospital	Jinan, Shandong Province, China	Patient recruitment	
Weidong	Qi		MD	Jinan People’s Hospital	Jinan, Shandong Province, China	Patient recruitment	
He	Meng		MD	Second Affiliated Hospital of Shandong University of Traditional Chinese Medicine	Jinan, Shandong Province, China	Patient recruitment	

Supplemental Online Content: Nonauthor Collaborators
*First name, last name, and suffix (if applicable) are required and will appear in PubMed.

***First Name and Middle Initial(s)**	***Last Name**	***Suffix (eg, Jr, III)**	Academic Degrees	Institution	Location (city, state/province, country)	Role or Contribution, eg, chair, principal investigator	Group (if more than 1 Group listed in the byline) and/or Subgroup (eg, Steering Committee)
Jin	Xuan		MD	Second Affiliated Hospital of Shandong University of Traditional Chinese Medicine	Jinan, Shandong Province, China	Patient recruitment	
Anjing	Ji		MD	Yuhuan Second People's Hospital	Taizhou, Zhejiang Province,China	Patient recruitment	
Wenjuan	Cai		MD	Changshu Hospital of Traditional Chinese Medicine	Suzhou, Jiangsu Province, China	Patient recruitment	
Yali	Gu		MD	Changshu Hospital of Traditional Chinese Medicine	Suzhou, Jiangsu Province, China	Patient recruitment	
Xiaowu	He		MD	The PLA Navy Anqing Hospital, Anqing	Anqing,Anhui Province, China	Patient recruitment	
