# Supplementary material for: Intracardiac vs Transesophageal Echocardiography in Atrial Fibrillation Ablation: A Randomized Clinical Trial
Source: JAMA Cardiol. 2025 Oct 8;10(12):1249–56. doi: 10.1001/jamacardio.2025.3687 (PMC12509080; doi:10.1001/jamacardio.2025.3687)
Supplement: Supplement 4. — Data Sharing Statement [file jamacardiol-e253687-s004.pdf]

Data Sharing Statement

Hu. Intracardiac vs Transesophageal Echocardiography in Atrial Fibrillation Ablation. *JAMA Cardiol*. Published October 08, 2025. doi:10.1001/jamacardio.2025.3687

Data
Additional Information: Clinical Trials, https://clinicaltrials.gov/, NCT05466266
Data available: Yes
Data types: Deidentified participant data
How to access data: To access these data, please send a request to drliuxu@126.com. Our team will process your request and provide further instructions for data access.
When available: With publication

Supporting Documents
Document types: Statistical/analytic code, Informed consent form
How to access documents: To access these Documents, please send a request to drliuxu@126.com.
When available: With publication

Additional Information
Who can access the data: Researchers whose proposed use of the data has been approved
Types of analyses: The deidentified participant data will be made available for the following types of analyses: For any purpose related to research in the field of atrial fibrillation.
Mechanisms of data availability: With investigator support: Our team will provide guidance and support to assist with data interpretation and usage. After approval of a proposal: Access to the data will be granted following the review and approval of a detailed research proposal outlining the intended use of the data. With a signed data access agreement: Researchers will be required to sign a data access agreement to ensure proper handling and confidentiality of the data. Priority for future analyses: The original study team retains priority for any subsequent subgroup or secondary analyses using these data. Access by external researchers will not affect ongoing analyses conducted by the original study team.
